# Type 1 diabetes in pregnancy is associated with distinct changes in the composition and function of the gut microbiome

**DOI:** 10.1186/s40168-021-01104-y

**Published:** 2021-08-06

**Authors:** Alexandra J. Roth-Schulze, Megan A. S. Penno, Katrina M. Ngui, Helena Oakey, Esther Bandala-Sanchez, Alannah D. Smith, Theo R. Allnutt, Rebecca L. Thomson, Peter J. Vuillermin, Maria E. Craig, William D. Rawlinson, Elizabeth A. Davis, Mark Harris, Georgia Soldatos, Peter G. Colman, John M. Wentworth, Aveni Haynes, Simon C. Barry, Richard O. Sinnott, Grant Morahan, Naiara G. Bediaga, Gordon K. Smyth, Anthony T. Papenfuss, Jennifer J. Couper, Leonard C. Harrison, on behalf of the ENDIA Study Group

**Affiliations:** 1 Walter and Eliza Hall Institute of Medical Research, Melbourne, VIC 3052 Australia; 2 Department of Medical Biology, University of Melbourne, Melbourne, VIC 3010 Australia; 3 The University of Adelaide, Robinson Research Institute, Adelaide Medical School, University of Adelaide, Adelaide, SA 5005 Australia; 4 Faculty of School of Medicine, Deakin University and Child Health Research Unit, Barwon Health, Geelong, VIC 3220 Australia; 5 School of Women’s and Children’s Health, Faculty of Medicine, University of New South Wales, Sydney, NSW 2052 Australia; 6 Institute of Endocrinology and Diabetes, The Children’s Hospital at Westmead, Sydney, NSW 2145 Australia; 7 Virology Research Laboratory, Serology and Virology Division, South Eastern Area Laboratory Services Microbiology, Prince of Wales Hospital, Sydney, NSW 2031 Australia; 8 School of Medical Sciences, Faculty of Medicine, University of New South Wales, Sydney, NSW 2052 Australia; 9 Telethon Institute for Child Health Research, Centre for Child Health Research, University of Western Australia, Perth, WA 6009 Australia; 10 The University of Queensland Diamantina Institute, Faculty of Medicine, University of Queensland, Translational Research Institute, Woolloongabba, QLD 4102 Australia; 11 Queensland Children’s Hospital, South Brisbane, QLD 4101 Australia; 12 Monash Centre for Health Research and Implementation, School of Public Health and Preventive Medicine, Monash University, Melbourne and Diabetes and Vascular Medicine Unit, Monash Health, Melbourne, VIC 3168 Australia; 13 Department of Diabetes and Endocrinology, Royal Melbourne Hospital, Melbourne, VIC 3050 Australia; 14 Melbourne eResearch Group, School of Computing and Information Services, University of Melbourne, Melbourne, VIC 3010 Australia; 15 Centre for Diabetes Research, Harry Perkins Institute of Medical Research, The University of Western Australia, Perth, WA 6009 Australia; 16 Department of Medical Biology and School of Mathematics and Statistics, University of Melbourne, Melbourne, VIC 3010 Australia; 17 Bioinformatics and Cancer Genomics Laboratory, Peter MacCallum Cancer Centre, Melbourne, VIC 3000 Australia; 18 Sir Peter MacCallum Department of Oncology, University of Melbourne, Parkville, VIC 3010 Australia; 19 Women’s and Children’s Hospital, Adelaide, SA 5006 Australia

**Keywords:** Microbiome, Gut, Type 1 diabetes, Pregnancy, Metagenomics, Quantitative PCR, Inflammation markers

## Abstract

**Background:**

The gut microbiome changes in response to a range of environmental conditions, life events and disease states. Pregnancy is a natural life event that involves major physiological adaptation yet studies of the microbiome in pregnancy are limited and their findings inconsistent. Pregnancy with type 1 diabetes (T1D) is associated with increased maternal and fetal risks but the gut microbiome in this context has not been characterized. By whole metagenome sequencing (WMS), we defined the taxonomic composition and function of the gut bacterial microbiome across 70 pregnancies, 36 in women with T1D.

**Results:**

Women with and without T1D exhibited compositional and functional changes in the gut microbiome across pregnancy. Profiles in women with T1D were distinct, with an increase in bacteria that produce lipopolysaccharides and a decrease in those that produce short-chain fatty acids, especially in the third trimester. In addition, women with T1D had elevated concentrations of fecal calprotectin, a marker of intestinal inflammation, and serum intestinal fatty acid-binding protein (I-FABP), a marker of intestinal epithelial damage.

**Conclusions:**

Women with T1D exhibit a shift towards a more pro-inflammatory gut microbiome during pregnancy, associated with evidence of intestinal inflammation. These changes could contribute to the increased risk of pregnancy complications in women with T1D and are potentially modifiable by dietary means.

**Video abstract**

**Supplementary Information:**

The online version contains supplementary material available at 10.1186/s40168-021-01104-y.

## Background

The gut microbiome provides essential metabolites, vitamins, co-factors and hormones, protects against pathogenic microorganisms and has a key role in the development of the immune and other systems [1, 2]. Changes in the composition of the gut microbiome are associated with ageing, environmental conditions, life events and disease states [2–4]. In pregnancy, women undergo significant physiological changes, but only recently has the gut microbiome been studied in this context [5, 6]. Koren et al. [5] sampled the gut microbiome in the first and third trimesters and found that the taxonomic composition in the first trimester was similar to that of non-pregnant women but in the third trimester the abundance of *Actinobacteria* and *Proteobacteria* phyla increased along with an overall decrease in bacterial richness (alpha diversity). In studies in germ-free mice, they observed that inoculation with third compared to first trimester feces led to greater weight gain, insulin resistance and gut inflammation and suggested this was an adaptive proinflammatory response to defend the fetus from pathogens and provide it with nutrients. In contrast, after analysing fecal samples weekly across pregnancy, DiGiulio et al. [6] found no significant temporal differences in diversity or composition of the gut microbiome. These contrary findings and the dearth of studies warrant further investigation of the gut microbiome in pregnancy.

Type 1 diabetes (T1D) is an autoimmune disease in which insulin-producing β cells in the islets of the pancreas are destroyed by T lymphocytes leading to insulin deficiency [7]. In pregnancy, T1D is associated with systemic and intra-uterine markers of sub-clinical inflammation and higher risks of complications for mother and fetus [8–10]. Alterations in the bacterial gut microbiome have been reported in T1D, mainly in children at high risk and at diagnosis (reviewed in [11], [12–17]). They include a decrease in alpha diversity (richness) [12–14] and in the abundance of lactate- and butyrate-producing and mucin-degrading bacteria [13–17], and an increase in the abundance of the Bacteroides genus [13, 14]. Functionally, these compositional changes are reflected by a decreased abundance of genes encoding related metabolic pathways and enzymes, e.g. butyryl-coenzyme A (CoA)-CoA transferase [15] and butyryl-CoA dehydrogenase for butyrate synthesis [16]. These changes are not necessarily specific for T1D but nevertheless, they may have clinical consequences, including in pregnancy. Gut butyrate is a key determinant of gut health and regulator of gene expression and homeostatic immunity [18–20]. It is the major energy source for the colonic mucosa, induces the synthesis of mucin and it promotes gut epithelial integrity, preventing ‘gut leakiness’. In the non-obese diabetic (NOD) mouse model of T1D, dietary butyrate supplementation promoted an increase in regulatory T cells and a decrease in the incidence of spontaneous diabetes [20]. Increased gut leakiness has been described in established T1D [21] and recently by ourselves in association with gut microbiome changes in children with islet autoimmunity who progressed to T1D [22]. Gut leakiness with translocation of toxins and dietary antigens into the blood may result in systemic inflammation, reported with T1D in pregnancy complicated by pre-eclampsia [10]. Because a consensus about the gut microbiome in pregnancy is lacking, even in the absence of T1D, we applied shotgun whole metagenomic sequencing (WMS) to analyse the gut microbiome across pregnancy in women with and without T1D participating in the Australia-wide Environmental Determinants of Islet Autoimmunity (ENDIA) study.

## Results

### Study population

**Fig. 1:**
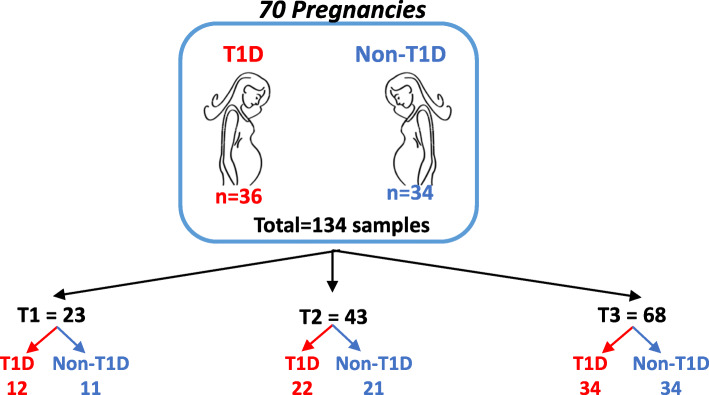
Fecal samples obtained in pregnancy. n: number of samples; T1: trimester 1; T2: trimester 2; T3: trimester 3; T1D: women with type 1 diabetes; Non-T1D: women without T1D

Fecal samples were collected between February 2013 and October 2017 from women enrolled in the ENDIA study, a prospective, pregnancy-birth cohort study that follows 1500 Australian children who have a first-degree relative with T1D [23]. Thirty-five women (36 pregnancies) with T1D and 31 women (34 pregnancies) without T1D had each provided from one to three fecal samples across pregnancy (total 134 samples) for analysis by shotgun WMS (Fig. 1). Table 1 summarizes and compares characteristics of the T1D and non-T1D pregnancies.

**Table 1:** Summary of characteristics of non-T1D and T1D pregnancies

General	Non-T1D	T1D	***P*** value**
Overall number of samples: *n* (%)	66 (49.3)	68 (50.7)	
Trimester 1	11 (16.7)	12 (17.6)	
Trimester 2	21 (31.8)	22 (32.4)	
Trimester 3	34 (51.5)	34 (50.0)	
All three trimesters (% pregnancies)	12 (35.3)	12 (33.3)	
All three trimesters (% samples)	12 (18.2)	12 (18.2)	
Gestational age in days at fecal sample: mean (SD)			
Trimester 1	75.4 (16.5)	75.3 (16.5)	
Trimester 2	150.8 (26.6)	148.4 (26.6)	
Trimester 3	247.6 (14.6)	234.7 (14.6)	0.001
**Maternal**			
Overall number of pregnancies	34	36	
Age in years at conception: mean (SD)	33 (4.1)	32.3 (4.0)	
Paternal missing *n* (%)	1 (2.9)	2 (5.6)	
Assisted conception: *n* (%)	3 (8.8)	4 (11.1)	
Twin pregnancy: *n* (%)	0 (0.0)	0 (0.0)	
Nulliparous: *n* (%)	14 (41.2)	18 (50.0)	
Pre-eclampsia: *n* (%)	0 (0.0)	4 (11.1)	0.018
Group B Streptococcus positive: *n* (%)	7 (20.6)	1 (2.8)	
Genito-urinary infections: *n* (%)	2 (5.9)	5 (13.9)	
Pre-pregnancy BMI: mean (SD)	24.7 (4.8)	25.8 (4.7)	
Underweight (< 18.5): *n* (%)	0 (0.0)	0 (0.0)	
Normal weight (18.5–24.9): *n* (%)	21 (61.8)	18 (50.0)	
Overweight weight (25–29.9): *n* (%)	6 (17.6)	10 (27.8)	
Obese (> 30): *n* (%)	7 (20.6)	8 (22.2)	
Gestational weight gain (kg): Mean (SD)	13 (5.0)	11.5 (4.9)	
Gestational weight gain (kg): *n* (%)	2 (5.9)	2 (5.6)	
**Paternal**			
Age in years at conception: mean (SD)	34.8 (5.2)	32.7 (4.8)	0.093
Pre-pregnancy BMI: mean (SD)	28.6 (4.3)	27.6 (4.3)	
Underweight (BMI < 18.5): *n* (%)	0 (0.0)	1 (2.8)	
Normal weight (BMI18.5–24.9): *n* (%)	4 (11.8)	6 (16.7)	
Overweight (BMI 25–29.9): *n* (%)	10 (29.4)	8 (22.2)	
Obese (> 30): *n* (%)	6 (17.6)	9 (25.0)	
Missing: n (%)	14 (41.2)	12 (33.3)	
**Maternal demographics**			
Born in Australia: n (%)			
Yes	30 (88.2)	24 (66.7)	
Unknown	0 (0.0)	1 (2.8)	
Education beyond high school: *n* (%)			
Yes	30 (88.2)	29 (80.6)	
Unknown	0 (0.0)	0 (0.0)	
Lives in a metro area: n (%)	31 (91.2)	35 (97.2)	
Socio-Economic Indexes for Areas (SEIFA) Index of Relative Socio-Economic Disadvantage (IRSD)			
Quintile 1 *n* (%)	2 (5.9)	1 (2.8)	
Quintile 2 *n* (%)	2 (5.9)	3 (8.3)	
Quintile 3 *n* (%)	13 (38.2)	8 (22.2)	
Quintile 4 *n* (%)	4 (11.8)	9 (25.0)	
Quintile 5 *n* (%)	13 (38.2)	15 (41.7)	
Smoking during pregnancy: *n* (%)	3 (8.8)	0 (0.0)	
Household smoking during pregnancy: *n* (%)	5 (14.7)	6 (16.7)	
Adults in house during pregnancy: *n* (%)			
One	0 (0.0)	2 (5.6)	
Two	31 (91.2)	29 (80.6)	
More than two	3 (8.8)	5 (13.9)	
Children in house during pregnancy: *n* (%)			
None	14 (41.2)	18 (50.0)	
One	8 (23.5)	10 (27.8)	
Two	5 (14.7)	7 (19.4)	
More than two	7 (20.6)	1 (2.8)	
Furred pet ownership during pregnancy: *n* (%)	24 (70.6)	20 (55.6)	
**Diet and physical activity in pregnancy**			
Diet: mean (SD)			
Energy/day (kJ)	6617.3 (2277.5)	6445.6 (2185.9)	
Fat (g)	68.8 (27.0)	71.7 (26.8)	
Protein (g)	77.1 (29.9)	80.3 (29.6)	
Carbohydrate (g)	163.8 (57.9)	142.4 (52.6)	
Fibre (g)	18.4 (6.1)	17.9 (5.9)	
Diet: Missing: *n* (%)	0 (0.0)	2 (5.6)	
Alcohol consumed: *n* (%)			
Yes	6 (17.6)	7 (19.4)	
Unknown	0 (0.0)	2 (5.6)	
Metabolic equivalent of task (MET) (h/wk): mean (SD)	254.5 (100.9)	267.9 (102.2)	
**Biological data**			
HbA1c (%)			
Trimester 1: median (IQR)	–	6.8 (1.6)	
Trimester 2: median (IQR)	–	6.1 (1.3)	
Trimester 3: median (IQR)	–	6.1 (0.8)	
Trimester 1: missing	–	1 (8.3)	
Trimester 2: missing	–	3 (13.6)	
Trimester 3: missing	–	14 (41.2)	
1,5-anhydroglucitol (AG) (μg/mL)			
Trimester 1: median (IQR)	14.1 (13.1)	3.4 (1.5)	
Trimester 2: median (IQR)	11.5 (4.9)	2.5 (2.3)	
Trimester 3: median (IQR)	8.1 (6.3)	2.4 (1.3)	
Trimester 1: mean (SD)	14.1 (5.9)	3.4 (2.9)	< 0.001
Trimester 2: mean (SD)	11.2 (5.1)	2.3 (2.2)	< 0.001
Trimester 3: mean (SD)	8.7 (3.8)	2.4 (2.0)	< 0.001
Trimester 2: missing n (%)	1 (4.8)	1 (4.5)	
Trimester 3: missing n (%)	2 (5.9)	8 (23.5)	
Serum vitamin D (nmol/L): mean (SD)			
Trimester 1	83 (26.9)	76.7 (25.8)	
Trimester 2	96.7 (27.0)	85.5 (24.5)	
Trimester 3	92.9 (31.6)	96.4 (29.9)	
Trimester 1: missing *n* (%)	0 (0.0)	1 (8.3)	
Trimester 3: missing *n* (%)	2 (5.9)	3 (8.8)	
Vitamin B6 (nmol/L): mean (SD)			
Trimester 3	76 (75.8)	70 (102.4)	
Vitamin B12 (nmol/L): mean (SD)			
Trimester 3	84 (116)	154 (138)	
Maternal HLA: n (%)			
DR34	3 (8.8)	14 (38.9)	
DR3 or DR4	20 (58.8)	19 (52.8)	
DRXX	11 (32.4)	3 (8.3)	0.002
**Known supplements in pregnancy**			
Antibiotics: *n* (%)	9 (26.5)	10 (27.8)	
Anticoagulants: *n* (%)	3 (8.8)	6 (16.7)	
Antihypertensive agents: *n* (%)	0 (0.0)	4 (11.1)	
**Known other supplements pre-pregnancy and pregnancy**			
Biotin: *n* (%)	12 (35.3)	9 (25.0)	
	29 (85.3)	30 (83.3)	
Calcium: *n* (%)	13 (38.2)	10 (27.8)	
	30 (88.2)	33 (91.7)	
Iron	14 (41.2)	9 (25.0)	
	32 (94.1)	35 (97.2)	
Magnesium: *n* (%)	14 (41.2)	9 (25.0)	
	31 (91.2)	32 (88.9)	
Selenium: *n* (%)	12 (35.3)	9 (25.0)	
	29 (85.3	30 (83.3)	
Vitamin B1: *n* (%)	14 (41.2)	9 (25.0)	
	32 (94.1)	32 (88.9)	
Vitamin B2: *n* (%)	14 (41.2)	9 (25.0)	
	31 (91.2)	32 (88.9)	
Vitamin B3: *n* (%)	14 (41.2)	9 (25.0)	
	31 (91.2)	32 (88.9)	
Vitamin B5: *n* (%)	9 (26.5)	6 (16.7)	
	18 (52.9)	16 (44.4)	
Vitamin B6: *n* (%)	14 (41.2)	9 (25.0)	
	32 (94.1)	32 (88.9)	
Vitamin B9 (folate): *n* (%)	14 (41.2)	15 (41.7)	
	32 (94.1)	36 (100.0)	
Vitamin B12: *n* (%)	14 (41.2)	9 (25.0)	
	32 (94.1)	30 (83.3)	
Vitamin D: *n* (%)	14 (41.2)	10 (27.8)	
	33 (97.1)	33 (91.7)	
Vitamin E: *n* (%)	13 (38.2)	8 (22.2)	
	28 (82.4)	27 (75.0)	
**Other**			
Vaccine: *n* (%)			
Yes (Flu only)	1 (2.9)	3 (8.3)	
Yes (Pertussis only)	3 (8.8)	3 (8.3)	
Yes (Flu and Pertussis)	11 (32.4)	10 (27.8)	
Mode of delivery: *n* (%)			
Vaginal	25 (73.5)	14 (38.9)	
Caesarean (with labour)	1 (2.9)	4 (11.1)	
Caesarean (without labour)	8 (23.5)	18 (50.0)	0.011

Log transformation was used for age at conception: Paternal

Square root transformation was used for all diet variables except carbohydrate and fibre

Square root transformation was used for 1,5-AG in all trimesters and for vitamin D in trimester 2

Hb1A-c, 1,5-AG and vitamin D are based on samples, not pregnancies

*NM* not measured

** Blank cells indicate *P* value non-significant. *P* values for HLA are determined against DRXX as baseline

### Whole metagenomic sequencing

The WMS dataset, 47,766,763 ± 10,956,057 (mean ± SD) paired-end reads per sample, was obtained using an Illumina NovaSeq 6000. Raw reads (SRA accession: PRJNA604850) were pre-processed using KneadData bioBakery tool [24] to eliminate human DNA sequences and filter sequences with poor quality which on average removed 6% of the reads. After quality control and read filter steps, 44,940,628 ± 10,572,188 (mean ± SD) paired-end reads per sample were obtained (Excel file E0).

### Taxonomic diversity and composition of the gut microbiome in women with and without T1D during pregnancy

Sequences were analysed with MetaPhLan2 implemented within the HUMAnN2 pipeline. Overall, 340 bacterial species were identified, with an average of 93 ± 13 (mean ± SD) species per sample. The top 25 most abundant species accounted for more than 50% of the gut microbiome composition of each subject in any given trimester (Figure S1).

Alpha diversity (observed richness or number of species) per sample was calculated and generalized estimating equations (GEE) were applied to test for differences between women without and with T1D, and between trimesters, and to determine if there was an interaction between T1D status and trimester. No differences were found in richness due to T1D status or time, or interactions (Figure S2, Excel file E1).

**Fig. 2:**
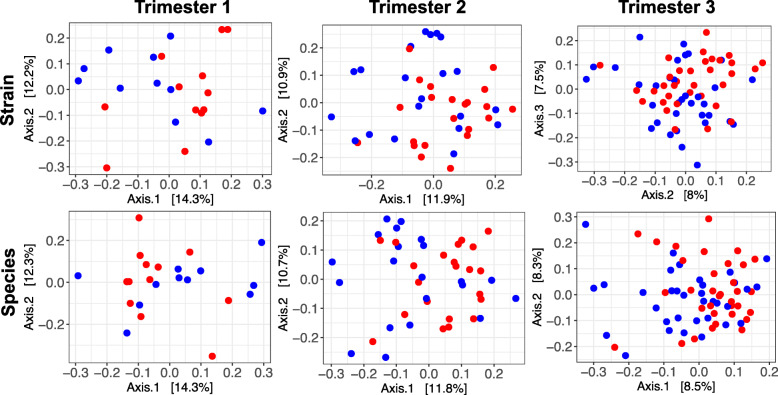
Beta diversity analysis by T1D status. PCoA ordination plots based on Bray-Curtis distances between samples at the strain and species taxonomic levels separated by trimesters in pregnancy. T1D: women with type 1 diabetes (red); Non-T1D: women without T1D (blue)

For analysis of beta diversity, Bray-Curtis coefficients were calculated between sample pairs, ordinated and plotted by principal coordinate analysis (PCoA) for each taxonomic level (Figs. 2, S3 and S4). To test for differences in beta diversity, a repeated-measure aware permutational analysis of variance (RMA-PERMANOVA) of the Bray-Curtis coefficients was performed on proportional log transformed data. This revealed a significant interaction between T1D status and time at all taxonomic levels. Therefore, differences between women with and without T1D were assessed within trimesters. No significant differences were detected in trimesters 1 and 2. However, differences were significant at the strain (*P* = 0.002), species (*P* = 0.001), genus (*P* = 0.070) and family (*P* = 0.034) levels in trimester 3 (Excel file E2).

To rule out the possibility that these results were influenced by the difference in sample size between trimesters 1 and 3, we performed a sensitivity analysis by subsampling trimester 3 to the size of trimester 1 (*n* = 23), using samples of trimester 3 from the same women in trimester 1, and repeated the beta diversity analysis. Similar to the complete trimester 3 dataset, differences were significant at the strain (*P* = 0.003), species (*P* = 0.003), genus (*P* = 0.043) and family (*P* = 0.047), but also phylum (*P* = 0.09), taxonomic levels (Excel file E2).

**Fig. 3:**
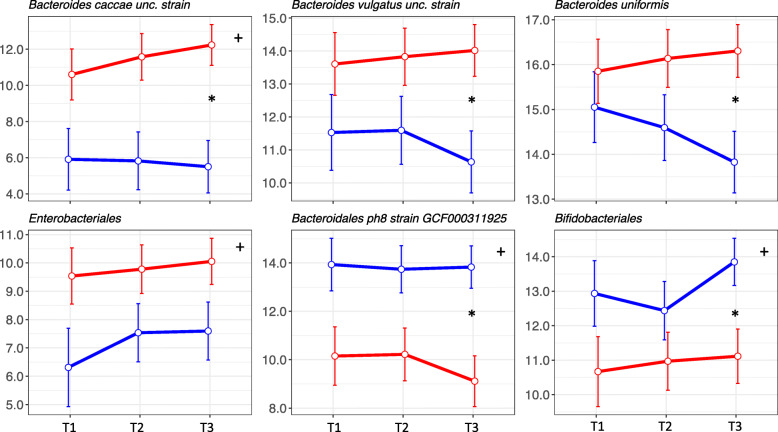
Means and standard errors of the log2-transformed fitted values shown as a point in each trimester for differentially abundant taxa in women with (red) and without (blue) T1D. * in the top right corner denotes a significant difference (FDR < 0.1) between groups throughout pregnancy; * between points denotes a significant difference (FDR < 0.1) between groups in that trimester

Differences in beta diversity reflect differences in taxonomic composition. To identify differences in specific taxa between women with and without T1D in pregnancy, differential abundance was analysed in limma. Only taxa for which the prevalence (i.e. proportion of samples with those taxa) was above 50% in at least one group and with a log2 fold-change (logFC) greater than 0.5 or less than − 0.5 were considered. Across all trimesters, the species *Bacteroides caccae* (FDR 0.03) and its unique strain (unclassified) in the dataset (FDR 0.03), as well as the order *Enterobacteriales* (FDR 0.07) were increased in women with T1D (Fig. 3; Excel file E3). On the other hand, species *Bacteroidales* bacterium ph8 (FDR 0.034) and its strain *(*GCF000311925) (FDR 0.03), the genus (FDR 0.08) and family (FDR 0.08) to which *Bacteroidales* bacterium ph8 belongs, and the order *Bifidobacteriales* (FDR 0.07), were decreased in women with T1D (Figure 3; Excel file E3).

Differences between women with and without T1D were also assessed within trimesters. In trimesters 1 and 2, taxa were not significantly different. However, several differences were found in trimester 3, in which the unique strain (unclassified) of *Bacteroides caccae* (FDR 0.004), the species *Bacteroides caccae* (FDR 0.004), the species *Bacteroides vulgatus* (FDR 0.04) and its unique strain (unclassified) (FDR 0.04) and *Bacteroides uniformis* (FDR 0.04) were increased in women with T1D, while the species *Bacteroidales* bacterium ph8 (FDR 0.01) and its strain *(*GCF000311925; FDR 0.005), and the genus (FDR 0.08) and family (FDR 0.08) of *Bacteroidales* bacterium ph8 and the order *Bifidobacteriales* (FDR 0.07), were decreased (Fig. 3; Excel file E3). A significant Spearman correlation (*R*^2^ > 0.4) was found between *B*. *caccae* and *B*. *vulgatus* (*R*^2^ = + 0.43; adj.*P* = 0.013).

A sensitivity analysis of differential abundance was also applied to the subset of trimester 3 samples referred to above: 13 species, 13 strains, 2 genera, 3 families, 2 orders and 3 phyla were detected as differentially abundant. From these, *Bacteroides caccae* and *Bacteroides uniformis*, an unclassified strain of *Bacteroides caccae* and the order *Bifidobacteriales* were also detected in the larger dataset of trimester 3 samples. Differential abundance results are summarised in Excel file E3.

In order to identify the bacterial species that were most abundant within the *Enterobacteriales* and *Bifidobacteriales* orders, we plotted the average relative abundance in women with and without T1D (Figure S5A). *Escherichia coli* was the most abundant species within *Enterobacteriales* and, together with an unclassified species of the genus *Escherichia*, accounted for almost the complete abundance of this order. In addition, a significant Spearman correlation was found between *E*. *coli* and *Coprococcus* sp. ART55_1 (*R*^2^ = − 0.6, adj.*P* = 0.09). *Bifidobacterium adolescentis* and *Bifidobacterium longum* were the most abundant species within *Bifidobacteriales* (Figure S5A). A lmer test applied to test differences in the abundance of these four species between women with and without T1D revealed that the abundance of *E*. *coli* in trimester 3 and of *B*. *adolescentis* in trimester 1 were significantly different between women with and without T1D (*P* = 0.01 for both; Figure S6).

### Effect of gestation time and other factors on the gut microbiome during pregnancy

No significant differences in alpha diversity were detected in women with or without T1D according to time, analysed either by days of gestation (*P* value 0.5) or by trimester (*P* values > 0.6), i.e. as continuous or categorical variables, respectively (Excel file E1). Due to the significant interaction between T1D status and time, differences in beta diversity across time (days or trimesters) were assessed separately in women with and without T1D (Excel file E2). Differences were detected only at the strain (*P* value 0.03) and species (*P* value 0.06) levels in women without T1D with time as continuous variable (Excel file E2). However, in women with T1D, differences in beta diversity across days of gestation and between trimesters were significant at all taxonomic levels except order and phylum (Excel file E2). These observations suggested that the microbial community structure across pregnancy is less stable in women with T1D. Therefore, we sought to identify differentially abundant taxa across trimesters separately within each group.

Throughout pregnancy, in women with T1D, the abundance of an unclassified species of the family *Peptostreptococcaceae* (FDR 0.02), the species *Odoribacter splanchnicus* (FDR 0.098), the genus *Prevotella* (FDR 0.066) dominated by the species *Prevotella copri* (Figure S5B) and the phylum *Verrucomicrobia* (FDR 0.043) decreased, while an unclassified strain of species *Streptococcus thermophilus* (FDR 0.099) and the species *Streptococcus thermophilus* (FDR 0.04) and family *Porphyromonadaceae* (FDR 0.092) increased (Excel file E4; Figure S7). In women without T1D, an *Anaerostipes hadrus* GCF000332875 strain (FDR 0.038) and species *Anaerostipes hadrus* (FDR 0.059), an unclassified strain of *Haemophilus parainfluenzae* (FDR 0.001) and species *Haemophilus parainfluenzae* (FDR 0.003), genus *Haemophilus* (FDR 0.004), family *Pasteurellaceae* (FDR 0.002), strain (GCF000218445 [FDR 0.04]) and species of *Lachnospiraceae* bacterium 1157FAA (FDR 0.055) and an unclassified species of *Veillonella* genus (FDR 0.083) decreased during pregnancy (Excel file E4; Figure S7). Furthermore, in women without T1D strains *Ruminococcus* sp. 5139BFAA GCF000159975 and *Lachnospiraceae* bacterium 3157FAACT1 GCF000218405 (FDR 0.063 and 0.075, respectively) and their corresponding species (FDR 0.065 and 0.075), unclassified strains of *Streptococcus thermophilus* (FDR 0.06) and *Bifidobacterium_animalis* (FDR = 0.06) and their corresponding species (FDR 0.06 for both) increased throughout pregnancy (Excel file E4; Figure S7).

As expected, women with and without T1D differed in serum 1,5-anhydroglucitrol (1,5-AG), a marker of short-term glycemic control [25] (Table 1), but in women with T1D, serum 1,5-AG was related to beta diversity only at the phylum level (Excel file E2). Mode of delivery had an effect on the beta diversity only at the family level (Excel file E2). No significant associations were found between beta diversity and age at conception, body mass index (BMI), parity, carbohydrate or fibre intake (Excel file E2). However, a difference was observed in the microbiome composition at the strain and species levels according to the human leukocyte antigen (HLA) class II type (Excel file E2). The model used to test for differences in beta diversity between women with and without T1D was adjusted for HLA type. HLA type accounted for 3.2% of the variation [*R*^2^] in beta diversity in trimester 3 (Excel file E2). After controlling for this effect, T1D status explained 2.9% of the variation and the difference in beta diversity between women with and without T1D women was statistically significant (*P* = 0.004) (Excel file E2). Finally, even though for the differential abundance analysis an adjustment for HLA type was included in the model, an additional analysis was performed to detect differences in the abundance of specific taxa due to HLA type and to verify that the taxa that were detected as differentially abundant due to T1D status were not affected by HLA type. Differences due to HLA type were detected only between HLADR34 and HLADR3X and DR4X for the abundance of strain *Eubacterium ramulus* GCF000469345 and species *Eubacterium ramulus* in trimester 1 and an unclassified strain of species *Eubacterium rectale* and species *Eubacterium rectale* in trimester 3, which were decreased in women with HLA DR34 Excel file E3. None of the taxa identified as differentially abundant due to T1D status were significantly affected by HLA type.

### Validation of differentially abundant species by qPCR

To validate the findings from metagenomic sequencing, we analysed the relative abundance of two of the top-ranked differentially abundant bacteria, *Bacteroides caccae* and *Bacteroides vulgatus*, in the same cohort of T1D and non-T1D mothers in trimester 3. Relative abundances obtained by metagenomic sequencing and qPCR were strongly correlated (Spearman *R* = + 0.91 and + 0.74 for *B*. *caccae* and *B*. *vulgatus*, respectively). By fitting linear models in lmer with conception age, BMI, parity and HLA type introduced as fixed effects, and ‘woman ID’ and processing batches as random effects, qPCR confirmed the increase in relative abundance of *B*. *caccae* (*P* = 0.00005) and *B*. *vulgatus* (*P* = 0.04) in women with T1D (Figure S8).

### Functional annotation of gut microbiome taxa

**Fig. 4:**
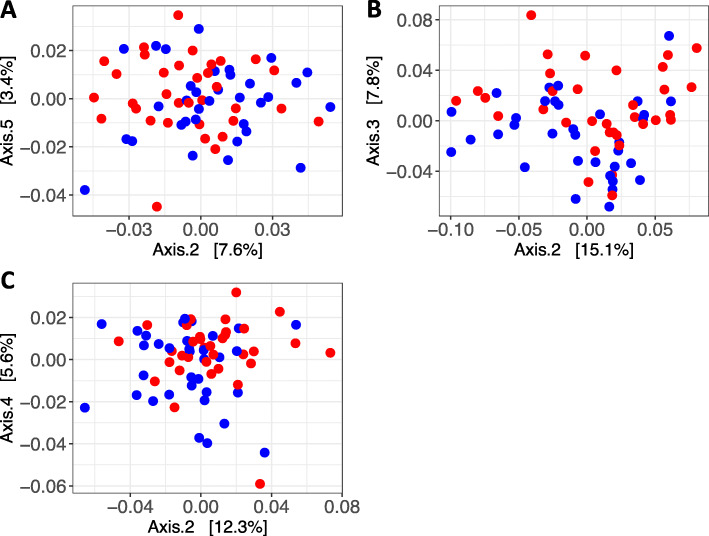
Beta diversity analysis by type 1 diabetes (T1D) status. PCoA ordination plots based on Bray-Curtis distances between samples for **a** pathways, **b** KOs and **c** MetaCyc reactions in trimester 3. T1D: women with type 1 diabetes (red); women without T1D (blue)

Sequences processed with HUMAnN2 were annotated, complete metabolic pathways quantified, gene abundances calculated and regrouped into KO (Kegg Orthology) and MetaCyc reaction functional categories. A total of 451 complete pathways, 5628 KO and 3204 MetaCyc reaction categories were obtained. No significant interaction in richness was detected between factors T1D status and time. In the model in which time was considered as a continuous variable, richness was significantly higher in women with T1D for all three functional categories (Figure S9, Excel file E1). For beta diversity, the interaction between T1D status and time was significant. Therefore, differences between groups were assessed within each trimester, but were significant for the three functional categories only in trimester 3 (Fig. 4; Excel file E2).

**Fig. 5:**
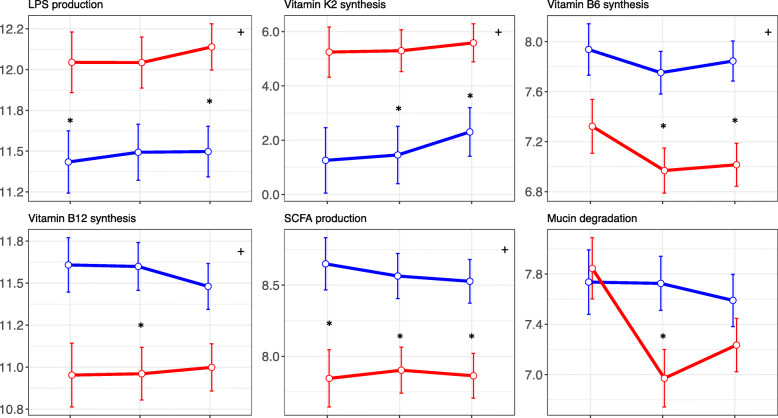
Means and standard errors of the log2-transformed fitted values shown as a point in each trimester for differentially abundant functional features in women with (red) and without (blue) T1D. One example for each of six broad categories is shown: lipopolysaccharide (LPS) production (CMP–3–deoxy–d–manno–octulosonate synthesis [PWY–1269]), vitamin K2 synthesis (superpathway of menaquinol–8 synthesis [PWY–5838]), vitamin B6 synthesis (pyridoxal 5′–phosphate synthase [K06215]), vitamin B12 synthesis (adenosylcobalamin salvage from cobinamide [COBALSYN–PWY]), short-chain fatty acid (SCFA) production (3–hydroxybutyryl–CoA dehydrogenase [K00074]) and mucin degradation (beta–*N*–acetylhexosaminidase [K01207]). * in the top right corner denotes a significant difference (FDR < 0.1) between groups throughout pregnancy; * between points denotes a significant difference (FDR < 0.1) between groups in that trimester

Women with and without T1D displayed significant differences in the abundance of a number of features identified in pathways, KO and MetaCyc categories; these are comprehensively listed in Supplementary Excel files E5-10. Selected functions, namely LPS production, vitamin K2 synthesis, vitamin B6 synthesis, vitamin B12 synthesis, short-chain fatty acid (SCFA) synthesis and mucin degradation, and the principal bacterial species contributing to these functions, are summarised in Table 2. Examples of bacteria contributing to a functional feature are shown in Figures S10 and S11. Of interest, a pathway (PWY1269: CMP-3-deoxy-d-manno-octulosonate biosynthesis I), 17 KO gene categories and two MetaCyc reactions (DARAB5PISOM-RXN and UDPGLCNACEPIM-RXN) involved in the synthesis of bacterial lipopolysaccharides (LPSs) were enriched in women with T1D (Excel file E5-E7; Table 2; Fig. 5, Figure S10A). Seven pathways and 6 KO categories involved in vitamin K2 synthesis were also increased in women with T1D (Excel files E5-E6; Table 2; Fig. 5; Figure S10B). In addition, two KO categories increased in women with T1D in trimester 3 were involved in antibiotic tolerance (K03771) and biofilm formation and (K18831) (Excel file E6).

**Table 2:** Pathways and enzymes differentially abundant in T1D women

Function (Change ref. T1D)	Function IDs	Principal bacterial contributors ^a^ (bacterial clusters are in bold)
LPS production (↑)	PWY-1269 K00748 K00912 K00979 K01447 K01627 K01791 K02517 K02536 K02852 K03270 K03771 K05807 K06041 K06142 K07091 K11720 K11934 DARAB5PISOM-RXN UDPGLCNACEPIM-RXN	***Escherichia coli, Akkermansia muciniphila, Bacteroides caccae, Alistipes finegoldii, Bacteroides dorei, Odoribacter splanchnicus, Alistipes shahii, Bacteroides fragilis, Bacteroides vulgatus, Bacteroides faecis, Bacteroides finegoldii, Bacteroides ovatus, Bacteroides sp. 2 1 22, Bacteroides sp. 4 3 47FAA, Bacteroides thetaiotaomicron, Bacteroides xylanisolvens, Alistipes onderdonkii, Bacteroides stercoris, Bacteroides uniformis****, Comamonas testosteroni, Enterobacter cloacae, Escherichia sp. TW09276, Achromobacter xylosoxidans, Aggregatibacter aphrophilus, Azospira oryzae, Bacteroides cellulosilyticus, Bacteroides sp. 1 1 6, Bacteroides sp. 3 2 5, Campylobacter concisus, Campylobacter curvus, Campylobacter hominis, Chryseobacterium taeanense, Citrobacter freundii, Citrobacter koseri, Cronobacter sakazakii, Delftia acidovorans, Desulfovibrio desulfuricans, Escherichia fergusonii, Eubacterium siraeum, Haemophilus influenzae, Neisseria flavescens, Neisseria meningitidis, Neisseria subflava, Parabacteroides goldsteinii, Parabacteroides merdae, Porphyromonas asaccharolytica, Prevotella denticola, Prevotella melaninogenica, Pseudomonas nitroreducens, Pseudomonas putida, Salmonella enterica, Serratia liquefaciens, Shigella flexneri, Shigella sonnei*
Vitamin K2 synthesis (↑)	PWY-5838 PWY-5845 PWY-5850 PWY-5860 PWY-5861 PWY-5862 PWY-5896 K00330 K00334 K00338 K00340 K00343 K02523 K02548	***Escherichia coli, Akkermansia muciniphila, Bacteroides sp. 4 3 47FAA, Bacteroides dorei, Bacteroides fragilis, Alistipes finegoldii, Bacteroides vulgatus****, Bacteroides salyersiae, Bacteroides sp. 1 1 6, Bacteroides thetaiotaomicron, Odoribacter splanchnicus, Alistipes onderdonkii, Alistipes shahii, Bacteroides caccae, Bacteroides ovatus, Bifidobacterium longum, Enterobacter cloacae, Klebsiella pneumoniae, Porphyromonas asaccharolytica*
Vitamin B6 synthesis (↓)	K06215	***Eubacterium rectale, Desulfovibrio piger***
Vitamin B12 synthesis (↓)	COBALSYN-PWY K02189 K03394 K05934 K05936 K06042 RIBAZOLEPHOSPHAT-RXN RXN-8770 2.7.1.156-RXN COBINAMIDEKIN-RXN COBINPGUANYLYLTRANS-RXN RXN-14063	***Eubacterium rectale, Faecalibacterium prausnitzii, Roseburia intestinalis, Desulfovibrio piger, Ruminococcus torques****, Ruminococcus obeum, Citrobacter freundii, Citrobacter koseri, Collinsella aerofaciens, Coprococcus catus, Fusobacterium nucleatum, Fusobacterium periodonticum, Klebsiella sp. MS 92 3, Lactobacillus reuteri, Megamonas funiformis, Megamonas hypermegale, Megamonas rupellensis, Methanosphaera stadtmanae, Morganella morganii, Roseburia hominis, Salmonella enterica, Streptococcus australis, Streptococcus parasanguinis, Streptococcus sanguinis, Veillonella dispar, Veillonella parvula, Veillonella sp. oral taxon 158*
SCFA production (↓)	GLUCUROCAT-PWY P42-PWY PWY-5177 PWY-6507 PWY-7242 K00016 K00074 K00248 K00626 K01571 K01625 K01715 K03856 K04070 K03785 K15634 METHYLACETOACETYLCOATHI OL-RXN RXN-12561 RXN0-2044 RXN-11245 RXN-16133 RXN-12705 RXN-14275 RXN-12750 RXN-12490 RXN-11662 RXN-12570 ACETYL-COA-ACETYLTRANSFER-RXN OHACYL-COA-DEHYDROG-RXN	***Faecalibacterium prausnitzii, Ruminococcus torques, Eubacterium rectale, Roseburia intestinalis, Anaerostipes hadrus, Lachnospiraceae bacterium 5 1 63FAA, Roseburia inulinivorans****, Roseburia hominis, Ruminococcus champanellensis, Bacteroides sp. 3 1 19, Bifidobacterium adolescentis, Bifidobacterium animalis, Eggerthella lenta, Eubacterium eligens, Eubacterium ventriosum, Ruminococcus bromii, Ruminococcus obeum, Treponema succinifaciens*
Mucin degradation (↓)	K01207	***Eubacterium rectale, Bifidobacterium adolescentis, Eubacterium siraeum, Ruminococcus bromii, Adlercreutzia equolifaciens, Roseburia intestinalis, Bifidobacterium bifidum, Streptococcus parasanguinis, Megamonas hypermegale***

^a^ On average more abundant in the group with increased abundance of a given function

The enzyme pyridoxal 5′-phosphate synthase (K06215) involved in the deoxyxylulose 5-phosphate (DXP)-independent pathway for vitamin B6 synthesis, one pathway, five KO categories and six metaCyc reactions related to vitamin B12 (cobalamin) synthesis and five pathways, 11 KO categories and 13 MetaCyc reactions involved in SCFA synthesis, including pyruvate and acetyl-CoA production and butyrate synthesis from acetate or lactate, were decreased in women with T1D (Excel file E5-E7; Table 2; Fig. 5; Figure S11). The abundance of beta-*N*-acetylhexosaminidase (K01207) involved in the degradation of mucin was also significantly reduced in women with T1D, but again only in trimester 2 (Table 2; Excel file E6; Fig. 5; Figure S11).

### Identification of bacterial clusters based on differentially abundant functional features

Differentially abundant functional features derived from HUMAnN2 were contributed not by a single species but rather a combination of species. Therefore, relative abundances of the principal contributing species in each of the six selected functions could be grouped into clusters (Table 2). For functions with three or more features, only principal contributors to at least three features were considered. For each cluster, a linear model was fitted with lmer using the same design as for the differential abundance analysis. This confirmed that women with T1D had an increased abundance of bacterial clusters contributing to production of LPS and synthesis of vitamin K2 and a decreased abundance of bacterial clusters contributing to synthesis of vitamins B6 and B12, production of SCFA and degradation of mucin (Figure S12).

### Markers of gut pathology

Because the composition and function of the gut microbiome of women with T1D was suggestive of a pro-inflammatory state, we sought evidence for gut inflammation in women with T1D. Fecal calprotectin, released from neutrophils and monocytes, is a marker of intestinal inflammation that may result in increased epithelial permeability [26]. Serum intestinal fatty acid-binding protein (I-FABP) is a marker of intestinal epithelial damage [27]. Fecal calprotectin and serum I-FABP were measured in trimester 3 in 61 women (32 with T1D) and 55 women (27 with T1D), respectively. Fecal calprotectin was increased in women with T1D compared to those without T1D (112 ± 148 vs. 36 ± 28 [mean ± SD] mg/kg: *P* value 0.04; Mann-Whitney test). Serum I-FABP was also increased in women with T1D compared to women without T1D (587 ± 235 vs. 314 ± 185 [mean ± SD] pg/mL: *P* value 0.0003; Mann-Whitney test) (Figure S13). However, these markers did not significantly correlate (Spearman *R* > 0.4) with any of the individual taxa that were differentially abundant between T1D and non-T1D women.

## Discussion

The gut microbiome in pregnancy has previously been analysed in two studies, by Koren et al*.* [5] and DiGiulio et al. [6], who pyrosequenced the 16S rRNA gene V1–V2 and V3–V5 regions, respectively, but with different conclusions. To our knowledge, the current study is the first to also include women with T1D, who have a higher frequency of complications and evidence of systemic and intra-uterine inflammation in pregnancy [8, 11, 12] that could conceivably be related to the gut microbiome. Koren et al. [5] compared single samples from trimesters 1 and 3 from 91 pregnancies and reported a decrease in alpha diversity and ‘remodelling of the gut microbiome’ by the third trimester, specifically a decrease in the abundance of taxa in the genus *Faecalibacterium* that generate the anti-inflammatory SCFA butyrate [18] and an increase in taxa in the phylum *Proteobacteria* recognised to be pro-inflammatory [28]. On the other hand, DiGiulio et al. [6] by weekly sampling of 49 women found no significant changes in diversity or composition across pregnancy. Similar to DiGiulio et al. [6], we observed no differences across pregnancy in alpha diversity but found differences in beta diversity at the strain and species levels in women without T1D and at all taxonomic levels in women with T1D. In addition, particularly in women with T1D, we saw changes in the relative abundance of specific taxa across pregnancy with progression to a more pro-inflammatory microbiome, similar to Koren et al. [5]. The taxonomic differences between women with and without T1D were reinforced by functional annotation, revealing differential abundance in enzymes and pathways as pregnancy progressed. These differences could not be attributed to demographic or other factors, including diet. It is important, however, to keep in mind that our findings are based on DNA analysis and they might not necessarily reflect changes at the RNA or protein level.

In examining differential abundance, we observed two main patterns: (1) taxa that were differentially abundant between women with and without T1D across all trimesters and (2) taxa that were similar in abundance in women with and without T1D in the first trimester but decreased or increased to be differentially abundant in trimester 3. In the first category, *B*. *caccae* and the order *Enterobacteriales* were increased in women with T1D across all trimesters. Within *Enterobacteriales*, *Escherichia coli* was the most abundant species and was enriched in women with T1D. Products of *E*. *coli* including lipopolysaccharide [29] and microcin [30] promote intestinal inflammation, intestinal permeability and low-grade systemic inflammation, and are implicated especially in the pathogenesis of inflammatory bowel disease [31, 32]. Moreover, an increase in these facultative anaerobes may displace obligate anaerobic bacteria that produce SCFAs [32], supported by the negative correlation between the abundance of *E*. *coli* and *Coprococcus* sp. ART55_1, further accentuating inflammation. In women with T1D, this may contribute to the decrease in abundance of the genus *Prevotella* comprising almost entirely *Prevotella copri*, a species that produces succinate and the SCFAs propionate and acetate known to be associated with improved glucose metabolism [19, 33]. Furthermore, in women with T1D, *E*. *coli* contributed to an increased abundance of enzymes involved in antibiotic tolerance (K18831) and biofilm formation (K03771). Bacterial biofilms confer increased tolerance to antibiotics and host immune responses [34] and may provide *E*. *coli* with a protective advantage over other more sensitive bacteria that compete for the same resources in the gut including SCFA-producers, which were less abundant in women with T1D.

In the second category (taxa that became differentially abundant by trimester 3), we observed that three species from the *Bacteroidales* order, *B*. *caccae*, *B*. *uniformis* and *B*. *vulgatus*, were increased in women with T1D. The genus *Bacteroides* was reported to be more abundant in children with islet autoimmunity compared to healthy controls [11, 12]. Bacteria from the *Bacteroides* (*B*. *caccae*, *B*. *vulgatus*, *B*. *uniformis*, *B*. *dorei*, *B*. *fragilis*, *B*. *faecis*, *B*. *finegoldii*, *B*. *thetaiotaomicron*, *B*. *xylanisolvens*, *B*. *stercoris* and *B*. *ovatus*) and *Alistipes* (*A*. *finegoldii*, *A*. *onderdonkii* and *A*. *shahii*) genera, all belonging to the *Bacteriodales* order, formed part of the LPS bacterial cluster which was enriched in women with T1D especially by trimester 3.

Twenty-nine functional features related to the production of SCFAs were decreased in women with T1D. The SCFAs bacterial cluster was composed of *Faecalibacterium prausnitzii*, *Eubacterium rectale*, *Anaerostipes hadrus*, *Lachnospiraceae*_*bacterium 5 1 63FAA*, *Ruminococcus torques*, *Roseburia intestinalis* and *Roseburia inulinivorans* all of which belong to the class *Clostridia* and are major butyrate producers [35]. Butyrate prevents gut inflammation and promotes gut barrier function [19]. In addition, the enzyme beta-*N*-acetylhexosaminidase (K01207), which degrades mucin [36], contributed by *Eubacterium rectale*, *E*. *siraeum*, *Ruminococcus bromii*, *Bifidobacterium adolescentis*, *B*. *bifidum* and *Roseburia intestinalis*, was decreased in women with T1D, but only in trimester 2. Degradation of mucins produces oligosaccharides, and acetate and propionate, which together then stimulate mucus production and enhance epithelial integrity [37], preventing ‘gut leakiness’ and translocation of toxins and dietary antigens into the systemic circulation. Because mucin degradation was lower in women with T1D, the stimulus to mucin production would be less. This would also be contributed to by the lower abundance of butyrate-producing bacteria observed in women with T1D. Thus, the gut microbiome in women with T1D exhibits pro-inflammatory features likely to be associated with low-grade systemic inflammation.

Women with T1D bacterial functions associated with vitamin K2 (menaquinone; MK-7) synthesis were increased and those associated with synthesis of the B-group vitamins B6 (pyridoxine) and B12 (cobalamine) were decreased. Mammals cannot synthesize these vitamins and must acquire them from the diet or gut microorganisms [38]. A small study based on metagenomic sequencing of the gut microbiome [39] observed a similar increase in the vitamin K2 superpathway (PWY-5838) in people with type 2 diabetes. Vitamin K2 is required for blood clotting and bone health [40] but why its synthesis by gut bacteria is increased in diabetes is unclear. All B-group vitamins contribute to regulation of immunity-inflammation and their deficiency has been associated with inflammatory disorders [41]. Vitamin B6 deficiency has been associated with inflammatory markers in population-based studies [42] and is reported to be common in T1D [43, 44]. Of interest therefore, we found that the key enzyme in B6 synthesis, pyridoxal 5′-phosphate synthase (K06215), was decreased across pregnancy in women with T1D. Vitamin B12 has several anti-oxidant properties [45] and is required for conversion of succinate to propionate by *Prevotella* [46]. The majority of women with and without T1D reported taking multi-B group vitamins from early pregnancy (Table 1) and in the third trimester plasma B6 and serum B12 did not differ significantly between women with and without T1D (Table 1). Nevertheless, the relative deficiency of these vitamin-synthesizing gut bacteria in women with T1D could contribute to other alterations in the gut microbiome, underscoring the importance of dietary supplementation in this group of women.

Our findings reveal that the composition of the gut microbiome not only changes across pregnancy but in a distinct way in women with T1D. By the third trimester, women with T1D exhibited a more pro-inflammatory and catabolic gut microbiome profile, reflected by an increase in LPS-producing bacteria and a decrease in SCFA-producing bacteria. These changes may account for the increase in calprotectin (marker of gut inflammation) and I-FABP (marker of gut epithelial integrity) we observed in women with T1D, known to be associated with impaired epithelial barrier function and leakage of LPS and other bacterial products leading to low-grade systemic inflammation. We suggest that systemic inflammation secondary to changes in the gut microbiome in T1D may contribute to the increased risk of pregnancy complications in T1D. Furthermore, a pro-inflammatory gut microbiome in the mother may impact the infant postnatally. In an elegant study in mice, Aguero et al. [47] found that transient exposure to an auxotrophic *E*. *coli* mutant in the intestine of germ-free mothers in pregnancy accentuated innate immune development in the intestine of their germ-free offspring. This effect was mediated by the transfer, in part via maternal antibodies, of a range of *E*. *coli* products across the placenta and in the mother’s serum and milk. Thus, with a single gut bacterium, the mother primed the immune system of her offspring, before their exposure to the external environment [47]. If T1D mothers with an increased abundance of *E*. *coli* and other LPS-producing gut bacteria better prime innate immunity in their offspring, this could protect against potentially diabetogenic infections in early life [48] and account for the lower risk of T1D in infants with a maternal compared to paternal proband [49].

## Conclusions

The gut microbiome changes across pregnancy but these changes are distinct in women with T1D. They include an increase in bacteria with pro-inflammatory properties, a decrease in bacteria with anti-inflammatory properties and a decrease in bacteria that synthesize essential vitamins, which together may lead to low-grade gut inflammation, epithelial barrier dysfunction, increased epithelial permeability and low-grade systemic inflammation. These are features of the gut microbiome ‘dysbiosis’ observed in a wide range of diseases [50], some of which have shown clinical benefit in response to probiotic and other dietary interventions. The relationship of these changes to the increased risk to mother and fetus in the T1D pregnancy requires further investigation. Intervention by dietary means to promote a less pro-inflammatory gut microbiome could potentially benefit both mother and fetus.

## Methods

### Participants and study design

This study involved 70 pregnancies in women participating in the ENDIA pregnancy-birth cohort study [23], 36 in women with established T1D on daily insulin treatment and 34 in healthy women with no history of gestational diabetes. The main criterion for participation in ENDIA was an unborn child with a first-degree relative with T1D. Table 1 provides summary statistics for participants, on 66 women with 70 pregnancies (four sibling pairs). Therefore, four women were included twice in the study population (each with two pregnancies). The unit of observation is the *pregnancy*, and therefore observations from the same mother but different pregnancies have been included as separate observations, as characteristics might change between pregnancies. Trimesters were categorised according to gestational age: T1 0–99 days; T2 100–196; T3 197–274.

Women provided written informed consent and were enrolled into the study between 2013 and 2016 at one of eight clinical sites. Up to three study visits occurred during pregnancy, ideally one in each trimester. The study was approved by a Human Research Ethics Committee (HREC) at each clinical site, with the Women’s and Children’s Health Network HREC in Adelaide acting as the lead HREC under the Australian National Mutual Acceptance Scheme (reference number HREC/16/WCHN/066). ENDIA is an observational study registered on the Australia New Zealand Clinical Trials Registry (ACTRN1261300794707).

Maternal and paternal demographics, medical history, past-pregnancy history, pre-pregnancy weight, assisted conception status and plurality of pregnancy were recorded at the first opportunity. Standardized questionnaires were offered at each pregnancy visit to record pregnancy complications, antibiotic and supplement usage, maternal and household smoking, household composition and pet ownership. Maternal diet during pregnancy was measured at the third trimester visit using a validated 74 item food frequency questionnaire, Dietary Questionnaire for Epidemiological Studies version 2 (DQESv2) [51]. Even though this was administered only in the third trimester, evidence for stability of dietary intake over the course of the pregnancy was obtained from a separate, purpose-built ENDIA Pregnancy Lifestyle Questionnaire administered before each of the three study visits during pregnancy. This assessed consumption of milk (dairy and non-dairy), caffeinated and decaffeinated tea and coffee, caffeine-containing soft drinks, dairy products, soy, gluten containing cereals (wheat, barley and rye) and non-gluten containing cereals (rice, corn and oats). Analysis across the study visits revealed that on 86% of occasions respondents reported either the same unit or within one-unit difference between visits 1–2, visits 1–3 and visits 2–3. Magnitude changes of four or five units were reported on < 2% of occasions. This supports the DQESv2 as being reflective of the whole pregnancy period. Women were advised to take multi B-group vitamin supplements from as early as possible in pregnancy.

### Sample collections and analyses

Serum 1,5-AG, a measure of glucose control in pregnancy [25], was measured by GlycoMark (Nippon Kayaku Co. Ltd., New York, NY, USA) in a single batch. Serum vitamin D3 was measured with a Liaison Analyser by the DiaSorin method (DiaSorin, Turin, Italy). Plasma vitamin B6 was measured by the Chromsystems HPLC-based assay (Chromsystems Instruments & Chemicals, Gräfelfing, Germany). Serum vitamin B12 was measured by the Abbot Architect Chemiluminescent Microparticle Immunoassay.

Fecal samples were collected in accordance with our validated collection-processing-storage method [52]. Briefly, samples were captured in a toilet using the Easy Sampler device (Co-Vertec Ltd, Waterlooville, UK) then transferred into a sterile 70 mL collection jar. Participants were instructed to store the sample in the refrigerator prior to transport to the laboratory in an insulated bag within 24 h. Samples were divided into aliquots with a sterile spatula in a Biosafety Level 2 cabinet, then stored at − 80°C. A total of 134 fecal samples were collected from the 70 pregnancies with either two or three samples collected longitudinally in each pregnancy (Fig. 1). DNA was extracted from fecal samples at the Walter and Eliza Hall Institute of Medical Research (WEHI) with the MoBio PowerSoil kit (MoBio Laboratories, Carlsbad, CA) as per manufacturer’s instructions.

Fecal calprotectin (micrograms per kg) was measured in 31 and 26 samples collected in the third trimester from women with and without T1D, respectively, by quantitative, enzyme-linked immunoassay (CALPROTMOslo, Norway) according to the manufacturer’s instructions. Human serum intestinal fatty acid binding protein (I-FABP) (picograms/mL) was measured in 26 and 26 samples collected in the third trimester from women with and without T1D, respectively, by a commercial ELISA kit (Enzyme-Linked Immunosorbent Assay; Hycult Biotech, the Netherlands) according to the manufacturer’s instructions.

HLA DR typing was performed on DNA in saliva collected with OG-500 Oragene DNA tubes (DNA Genotek, Ontario, Canada) by TaqMan-based PCR-typing and imputation from three single-nucleotide polymorphisms (rs3104413, rs2187668 and rs9275495), as described previously [53].

### Whole metagenome sequencing and generation of taxonomic and functional profiles

Whole metagenome sequencing (WMS) libraries were generated and sequenced with the 2 × 150 bp paired-end chemistry on two separate runs of an Illumina NovaSeq 6000 (Illumina, San Diego, California, USA) sequencer at the Ramaciotti Centre for Genomics (UNSW, Sydney, Australia. www.ramaciotti.unsw.edu.au). Sequencing data were quality controlled, and reads aligning to the human genome were removed using KneadData (v0.6.1) [24]. For the functional analysis, filtered reads classified using Kraken2 with the standard database [54], were further processed using HUMAnN2 (v0.11.1) [55] with the UniRef90 database to generate functional annotations (i.e. genes and metabolic pathways) and define the metabolic potential of the microbial communities. A functional profile (i.e. function-per-sample counts matrix) for metaCyc [56] complete metabolic pathways was obtained. In addition, two functional profiles were generated by grouping genes into KO [57] and MetaCyc-reactions functional categories using the humann2_regroup_table command. As part of the HUMAnN2 pipeline, MetaPhLan2 (v 2.7.5) [58] was used on the reads filtered with KneadData, to detect and quantify individual species with a library of clade-specific markers (ChocoPhlAn database) and generate whole-metagenome-based profiles at strain, species, genus, family, order and phylum taxonomic levels. Taxonomic and functional profiles were imported into the phyloseq [59] package in R [60]. An abundance filter was applied to remove all taxa and functional categories with a relative abundance across all samples of < 0.01%.

Alpha diversity (diversity within microbial communities) was obtained from the number of observed taxa (richness) using the function estimate richness from the R package phyloseq.

Beta diversity (diversity between microbial communities) was determined with phyloseq (function distance, method=‘bray’) on proportional log transformed data. This function calculates Bray-Curtis coefficients, which measure the distance between communities based on the taxa/functions that they contain and their abundances. The data were visualised using principal coordinates analysis (PCoA) plots in phyloseq.

### Statistical analyses

For continuous responses, where appropriate, the summary tables present mean and standard deviation derived from fitting a linear mixed model. The model fit for each continuous response adjusts for the fact that the observations from women with more than one pregnancy are not fully independent but may be correlated. For some response variables, the assumption of normally distributed residuals was not met. In these analyses, the response variable was transformed using a square root or log transformation, as appropriate. For transformed responses, the back transformed means and approximated standard deviations are presented. A Wald’s test is used to determine whether the groups are significantly different.

For categorical responses, summary tables show numbers and percentages. The percentage was calculated using the total number of pregnancies or samples as the denominator. To determine whether the distribution of observations between groups for categorical data were similar or not, a generalized linear mixed model was fitted, with a random effect for woman (i.e. subject). Such models adjust for correlated women observations. To determine whether groups were significantly different, the change in deviation of the final mode (i.e. a likelihood ratio test), which includes and excludes the treatment term, was examined. A pre-set *P* value of 0.05 was used as a cut-off for determining statistical significance for all models. Data analysis was performed in R (R Core Team, 2018 [61];), with the R packages lme4 v1.1.21 [62], car v3.0.3 [63], predictmeans v1.0.4 [64] and nnet v7.3.12 [65].

For testing differences in alpha diversity between groups of interest, GEEs [66] were applied using the R function geeglm from package geepack v1.2-1 ([67]; parameter family set to default ‘Gaussian’) to account for possible correlation of multiple measurements within a woman over time. The default empirical (robust or ‘sandwich’) estimator was used to ensure that estimates are robust to misspecification of the correlation structure. The model used for the regression included T1D status and time (i.e. two models were tested considering time as a continuous [gestational days] or categorical [trimesters] variable) as well as their interaction term (T1D × days or trimester; to test if differences in alpha diversity between women with and without T1D change across days or trimesters) and was adjusted for sample processing batches (which includes sequencing run), conception age, BMI, parity and HLA type. Mean-centred values were used for gestational days, conception age and BMI to ensure that the model coefficients are meaningful.

Differences in beta diversity were evaluated by PERMANOVA using Bray-Curtis dissimilarities with the Adonis function from the vegan [68] R package. For tests that included multiple samples across trimesters from the same participant (i.e. longitudinal analysis), a modified version of Adonis, which performs a RMA-PERMANOVA test [32], was employed. This statistical model included T1D status and time with their interaction adjusted as in the alpha diversity model (i.e. adjusting for sequencing run, conception age, BMI, parity and HLA type). In addition, interactions between time and other factors were also tested as described in the results section. When an interaction was significant (i.e. FDR < 0.1), statistical analysis was performed within trimester (i.e. when testing for differences between women with and without T1D) or by separating data from women with and without T1D (i.e. when testing for differences in time).

Differential abundance of taxa from MetaPhlan2 and gene categories and metabolic pathways from HUMAnN2 was analysed with the R package limma [69]. First, taxonomic relative abundances from MetaPhlan were multiplied by the library size of each sample, whereas for the functional analysis of data generated with HUMAnN2 counts (CPM) were used. Taxonomic and functional data were filtered using the filterByExpr function with default parameters with an additional general abundance filter that removed all those taxa or functions with a relative abundance across all samples of < 0.01% and a prevalence filter that removed those taxa present in less than 33 samples (i.e. ~ 25% of the samples). Library sizes were normalized using the trimmed mean of log expression ratios with singleton pairing (TMMwsp) method [70] in edgeR which is expected to perform better for data with a high proportion of zeros. Counts were transformed to log2-counts per million (logCPM), voom precision weights were calculated and limma linear models were fitted while allowing for loss of residual degrees of freedom due to exact zeros using the voomLmFit function [71] [72]. Here, ‘women IDs’ were considered as blocks to calculate the consensus correlation and account for multiple measurements while estimating contrasts statistics using the contrasts.fit function and empirical Bayes moderated *t* statistics. Since we have samples for all possible combinations of T1D status and trimester, this is a factorial design. Therefore, in order to build our model, factors T1D status and trimester were combined into a single factor with six levels and the comparisons of interests were defined as contrasts. In addition, the model design included an adjustment for sample processing batches, conception age, BMI, parity and HLA type. For this, groups means were computed by mean-correcting covariates and factors before performing the test: for numerical covariates, the mean was subtracted and for factors the contr.sum function was used (e.g. contrasts(ExtractionBatch) <- contr.sum(levels(ExtractionBatch). The mean abundance of each taxon in each group is computed by limma when fitting the model and are contained in the coefficients component (fit$coef). The standard errors are obtained as following: fit$stdev.unscaled[,] * sqrt(fitc$s2.post). Note that after normalization, the data are on the log2 scale. For testing for differences in specific taxa due to HLA type, the limma model included, similarly to the previous model, ‘women IDs’ as blocks and adjustments for sample processing batches, conception age, BMI and parity. Four contrasts were fitted: (1) High (DR34) vs. low (DRXX, DR3X and DR4X) risk HLA types, (2) DR34 vs. DR3X and DR4X grouped into a factor, (3) DRXX vs. DR3X and DR4X grouped into a factor and (4) DR34 vs. DRXX. *P* values were adjusted with the Benjamini and Hochberg method to control the FDR. FDR < 0.1 were considered significant. Taxa or functions significantly different with an abundance logFC greater than 0.5 or less than − 0.5 and present in at least 50% of the samples in either of the groups being compared were regarded as biologically significant. For identifying the ‘principal bacterial contributors’ to each differentially abundant function, first, the HUMAnN2-generated files with functions (Kegg orthology, MetaCyc reaction and complete pathways) stratified by contributing species were obtained. Next, the functions of interest with contributing species were disaggregated into individual files using grep. Finally, limma was applied as explained above to each subset of function with contributing species and only species with a larger log2-FC in the group of interest for the specific function were considered principal contributors belonging to the same bacterial cluster. The significance of the difference between measurements of serum markers was tested with a Wilcoxon rank sum test equivalent to the Mann-Whitney test using the wilcox.test function from the stats R package with parameter paired set to FALSE.

### Real-time quantitative PCR analysis

The qPCR reaction comprised 10 μL Sybr Green GoTaq qPCR Master Mix (2×) (Promega), 0.3 mM of each primer, 8.4 μL of water and 1 ng of DNA in 20 μL. Assays were performed in triplicate using the QuantStudio 12K Flex Real-Time PCR System (Thermofisher) with the following protocol: one cycle at 95 °C for 10 min, followed by 40 cycles of a two-stage temperature profile at 95 °C for 15 s and 60 °C for 1 min. Primers were *Bacteroides vulgatus* (BV-1) 5′-GCATCATGAGTCCGCATGTTC-3′, BV-2 5′-TCCATACCCGACTTTATTCCTT-3′; Bacteroides caccae (BaCA-1) 5′-GGGCATCAGTTTGTTTGCTT-3′, BaCA-2 5′-GAACGCATCCCCATCTCATA-3′; universal 16S V4 primers Univ-1 5′-GTGYCAGCMGCCGCGGTAA-3′, Univ-2 5′-GGACTACNVGGGTWTCTAAT-3′. Standard curves were generated by 2-fold dilutions ranging from 10 to 0.02 ng of a pooled human fecal DNA.

Data from each triplicate fell within a 0.5 threshold cycle (Ct); outliers (> 1 standard deviation) were removed before calculating the average Ct of each sample. Amplification efficiency (E) was determined from the slope of the standard curves for each primer pair using the formula *E* = (10−1/slope)-1. Efficiencies ranged from 97 to 102%. The abundances (*N*) for *Bacteroides vulgatus* and *Bacteroides caccae* were determined relative to the total bacterial load measured with the universal 16S primers, where *N* (*B*. *vulgatus*) = (Efficiency_*B*. *vulgatus* + 1)^Ct_*B*.*vulgatus*^, *N* (*B*. *caccae*) = (Efficiency_*B*. *caccae* + 1)^Ct_*B*.*caccae*^, *N* universal = (Efficiency_16S universal + 1)^Ct_Universal^, *B*. *vulgatus* relative abundance = N (*B*. *vulgatus*)/ N Universal, *B*. *caccae*_relative abundance = N (*B*. *caccae*)/ N Universal.

Relative abundances were log10-transformed and used as input for the regression models. The association between the relative abundance of *Bacteroides caccae* and *Bacteroides vulgatus* and T1D status was determined using a linear mixed effects model (lmer) with conception age, BMI, parity and HLA type as fixed effects, and ‘woman ID’ and processing batches as random effects.

## Supplementary Information

**Additional file 1: Figure S1**. Taxonomic composition of the 25 most abundant species as measured by WMS in fecal samples collected in trimesters 1, 2 and 3 of 70 pregnancies from 66 women (35 with T1D). X-axis depicts the non-informative study ID in the format womanID_pregnancy number_trimester. LCBD: local contribution to beta diversity (a measure of the uniqueness of communities). T1D: women with type 1 diabetes, non-T1D: women without T1D. **Figure S2**. Alpha diversity (Richness), by T1D status of women across trimesters. **Figure S3**. Beta diversity analysis by T1D status of women. PCoA ordination plots based on Bray-Curtis distances between samples at the Genus and Family taxonomic levels separated by trimesters in pregnancy. **Figure S4**. Beta diversity analysis by T1D status of women. PCoA ordination plots based on Bray-Curtis distances between samples at the Order and Phylum taxonomic levels separated by trimesters in pregnancy. **Figure S5**. Relative abundance and species composition of A) two orders differentially abundant between women with and without T1D, and B) one genus differentially abundant between trimesters in women with T1D. **Figure S6**. Log2 transformation of the relative abundance (1000 + 0.01) of bacterial species within the *Enterobacteriales* and *Bifidobacteriales* orders in women with and without T1D (mean ± SEM). P: *P*-value. **Figure S7.** Taxa differentially abundant between trimesters in women with and without T1D (mean ± SEM). Transformed data are on a log2 scale with the mean from fitted abundances shown as a point in each trimester for each group of women. The between trimesters denotes a significant difference between those trimesters while the after the trend line denotes significant difference between trimesters 1 and 3. The color denotes if differences between trimesters are within women with or without T1D. **Figure S8**. Boxplots representing the abundance distribution obtained by real-time quantitative PCR (qPCR) in women and with without T1D. P: *P*-value. **Figure S9.** Boxplots representing the functional alpha diversity distribution in women with and without T1D). P: *P*-value. **Figure S10**. Taxa contributing to pathways that are differentially more abundant in women with compared to women without T1D and non-T1D. A) PWY1269: CMP-3-deoxy-D-manno-octulosonate pathway I involved in LPS biosynthesis, and B) PWY−5838: Superpathway of menaquinol−8 synthesis involved in vitamin K2 synthesis. Y-axis: log2 of CPM (counts per million). **Figure S11**. Taxa contributing to functional features that are differentially more abundant in women with compared to women without T1D. A) Pyridoxal 5'-phosphate synthase (K06215) involved in vitamin B6 synthesis, B) COBALSYN−PWY: Adenosylcobalamin salvage from cobinamide involved in vitamin B12 synthesis, C) 3−hydroxybutyryl−CoA dehydrogenase (K00074) involved in short chain fatty acid (SCFA) production, and D) beta−N−acetylhexosaminidase (K01207), only differentially abundant in trimester 2, involved in mucin degradation. Y-axis: log2 of CPM (counts per million). **Figure S12**. Log2 transformed relative abundance (+ 0.01) of bacterial clusters based on broader functions in women with and without T1D across trimesters. P: *P*-value. **Figure S13**. Boxplots representing the distribution of measures for calprotectin and I-FABP in women with and without T1D. P: *P*-value.

**Additional file 2.**

## Data Availability

The demultiplexed raw datasets supporting the conclusions of this study can be accessed in the NCBI SRA https://www.ncbi.nlm.nih.gov/sra (project number PRJNA604850). All the python commands used to run HUMAnN2 and the R code used to perform statistical analyses are available at GitHub (https://github.com/PapenfussLab/RothSchulze_pregnancy-gut-microbiome-T1D) as R markdown coding and knitr html files along with the necessary R objects which contain taxonomic and functional profiles with metadata.

## Abbreviations

AG: Anhydroglucitrol
ASV: Amplicon sequence variants
BMI: Body mass index
BCAA: Branched chain amino acid
CoA: Coenzyme A
CPM: Counts per million
ENDIA: Environmental determinants of islet autoimmunity
FDR: False discovery rate
GEE: Generalized estimating equations
HLA: Human leukocyte antigen
HPLC: High-performance liquid chromatography
HREC: Human research ethics committee
KO: Kegg orthology
LCBD: Local contribution to beta diversity
LogFC: Log2 fold-change
LPS: Lipopolysaccharide
NOD: Non-obese diabetic
OTU: Operational taxonomic unit
PCoA: Principal components analysis
PWY: Pathway (Metacyc)
RMA-PERMANOVA: Repeated measure-aware permutation analysis of variance
SCFA: Short-chain fatty acid
SD: Standard deviation
T: Trimester
TCA: Tricarboxylic acid
T1D: Type 1 diabetes
TMM: Trimmed mean of log expression ratios
WEHI: Walter and Eliza Hall Institute of Medical Research
WMS: Whole metagenomic sequencing
