# Supplementary material for: Type 1 diabetes in pregnancy is associated with distinct changes in the composition and function of the gut microbiome
Source: Microbiome. 2021 Aug 6;9:167. doi: 10.1186/s40168-021-01104-y (PMC8349100; doi:10.1186/s40168-021-01104-y)
Supplement: Supplementary file 2 — Additional file 1: Figure S1. Taxonomic composition of the 25 most abundant species as measured by WMS in fecal samples collected in trimesters 1, 2 and 3 of 70 pregnancies from 66 women (35 with T1D). X-axis depicts the non-informative study ID in the format womanID_pregnancy number_trimester. LCBD: local contribution to beta diversity (a measure of the uniqueness of communities). T1D: women with type 1 diabetes, non-T1D: women without T1D. Figure S2. Alpha diversity (Richness), by T1D status of women across trimesters. Figure S3. Beta diversity analysis by T1D status of women. PCoA ordination plots based on Bray-Curtis distances between samples at the Genus and Family taxonomic levels separated by trimesters in pregnancy. Figure S4. Beta diversity analysis by T1D status of women. PCoA ordination plots based on Bray-Curtis distances between samples at the Order and Phylum taxonomic levels separated by trimesters in pregnancy. Figure S5. Relative abundance and species composition of A) two orders differentially abundant between women with and without T1D, and B) one genus differentially abundant between trimesters in women with T1D. Figure S6. Log2 transformation of the relative abundance (1000 + 0.01) of bacterial species within the Enterobacteriales and Bifidobacteriales orders in women with and without T1D (mean ± SEM). P: P-value. Figure S7. Taxa differentially abundant between trimesters in women with and without T1D (mean ± SEM). Transformed data are on a log2 scale with the mean from fitted abundances shown as a point in each trimester for each group of women. The between trimesters denotes a significant difference between those trimesters while the after the trend line denotes significant difference between trimesters 1 and 3. The color denotes if differences between trimesters are within women with or without T1D. Figure S8. Boxplots representing the abundance distribution obtained by real-time quantitative PCR (qPCR) in women and with without T1D. P: P-val [file 40168_2021_1104_MOESM2_ESM.pptx]

## Slide 1
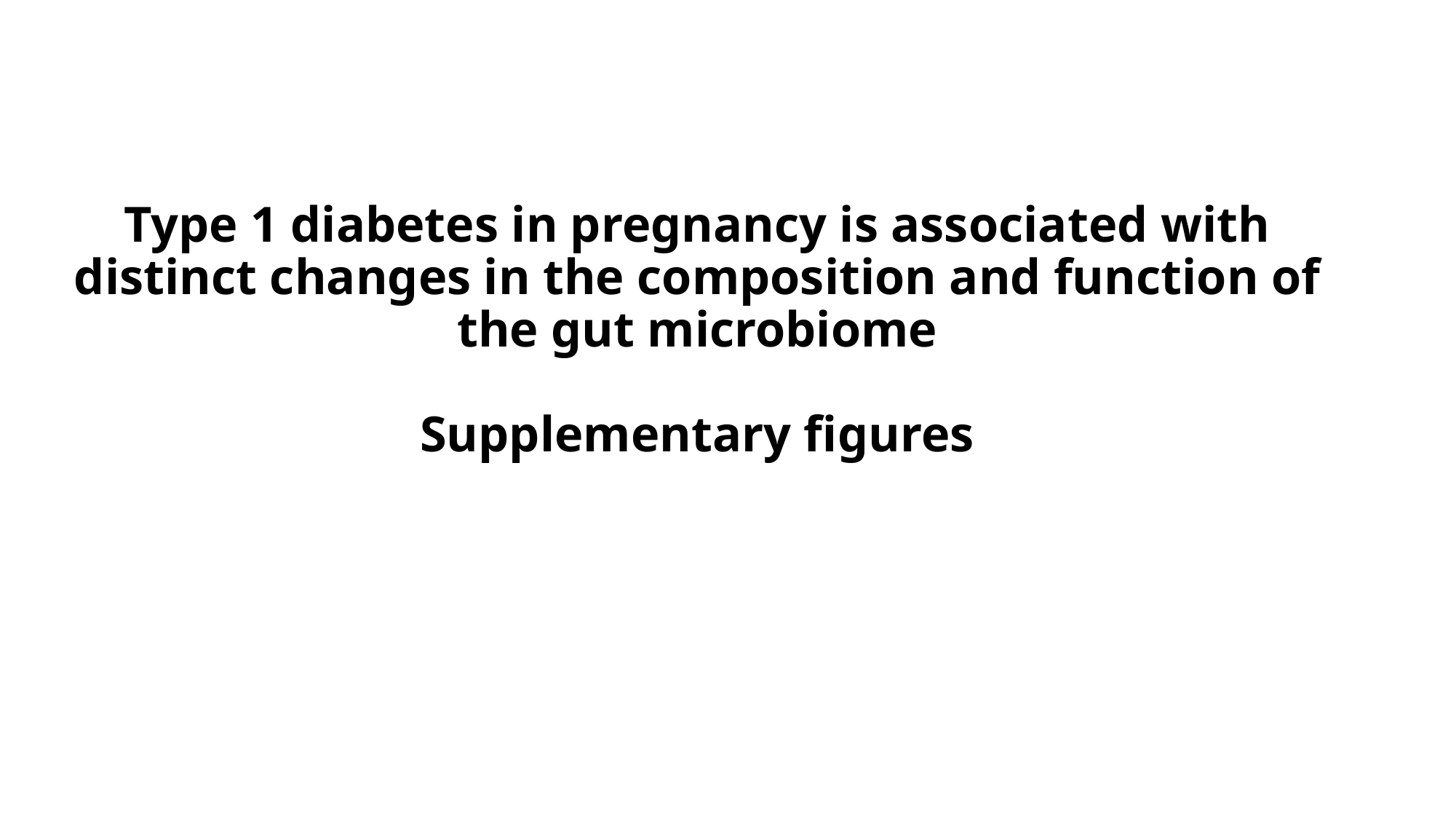

Type 1 diabetes in pregnancy is associated with distinct changes in the composition and function of the gut microbiome
Supplementary figures

## Slide 2
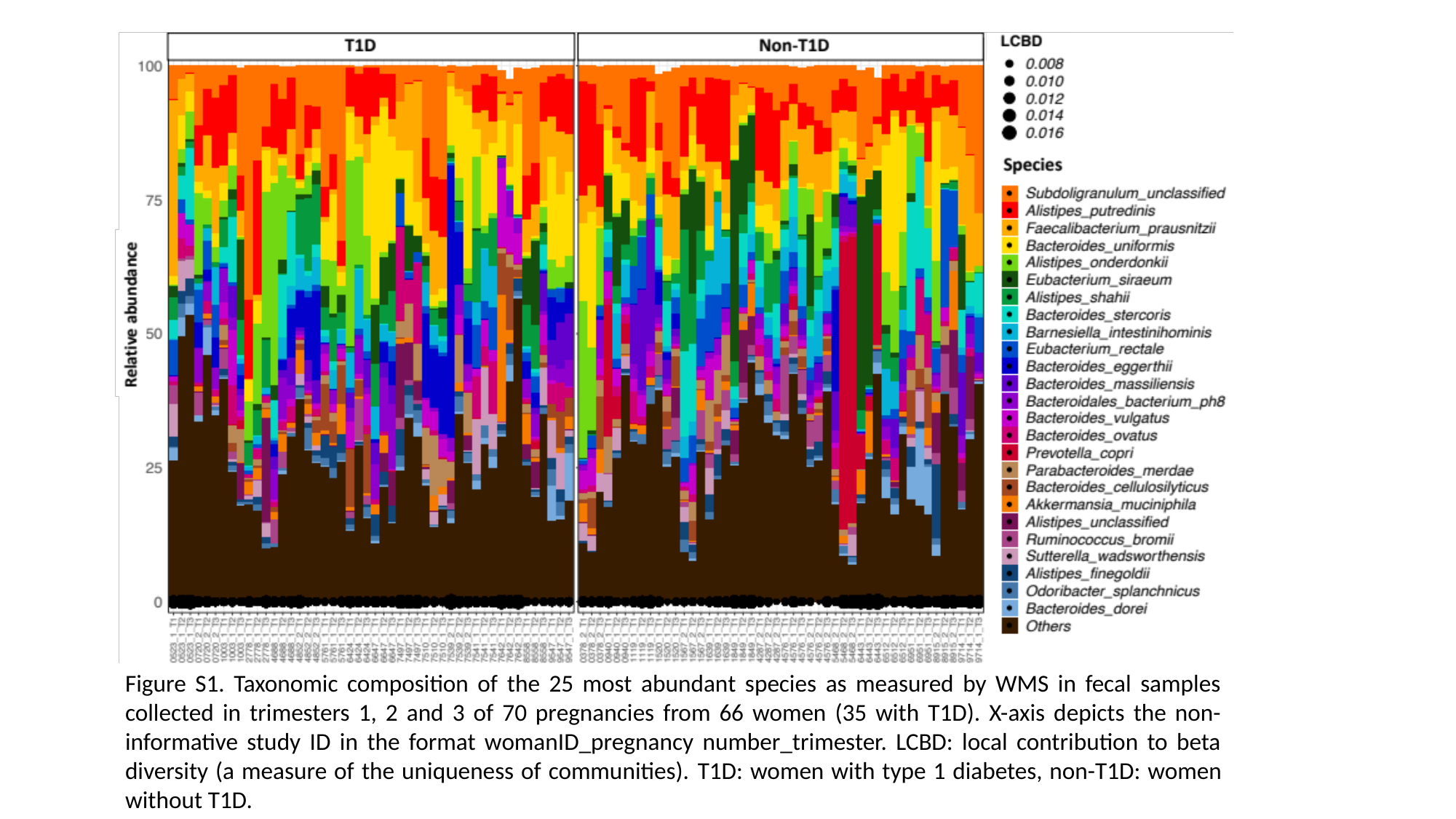

Figure S1. Taxonomic composition of the 25 most abundant species as measured by WMS in fecal samples collected in trimesters 1, 2 and 3 of 70 pregnancies from 66 women (35 with T1D). X-axis depicts the non-informative study ID in the format womanID_pregnancy number_trimester. LCBD: local contribution to beta diversity (a measure of the uniqueness of communities). T1D: women with type 1 diabetes, non-T1D: women without T1D.

## Slide 3
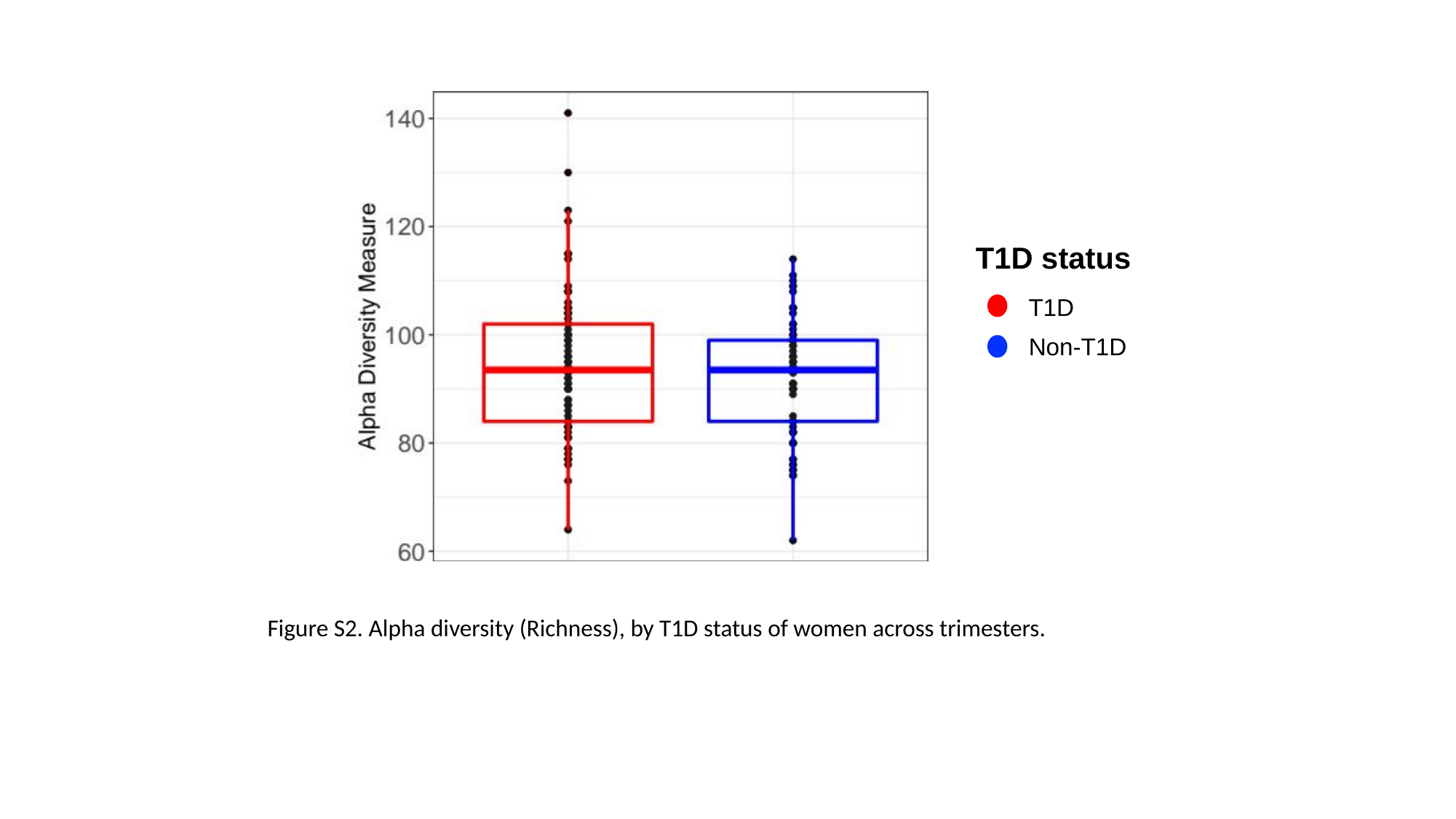

T1D status
T1D
Non-T1D
Figure S2. Alpha diversity (Richness), by T1D status of women across trimesters.

## Slide 4
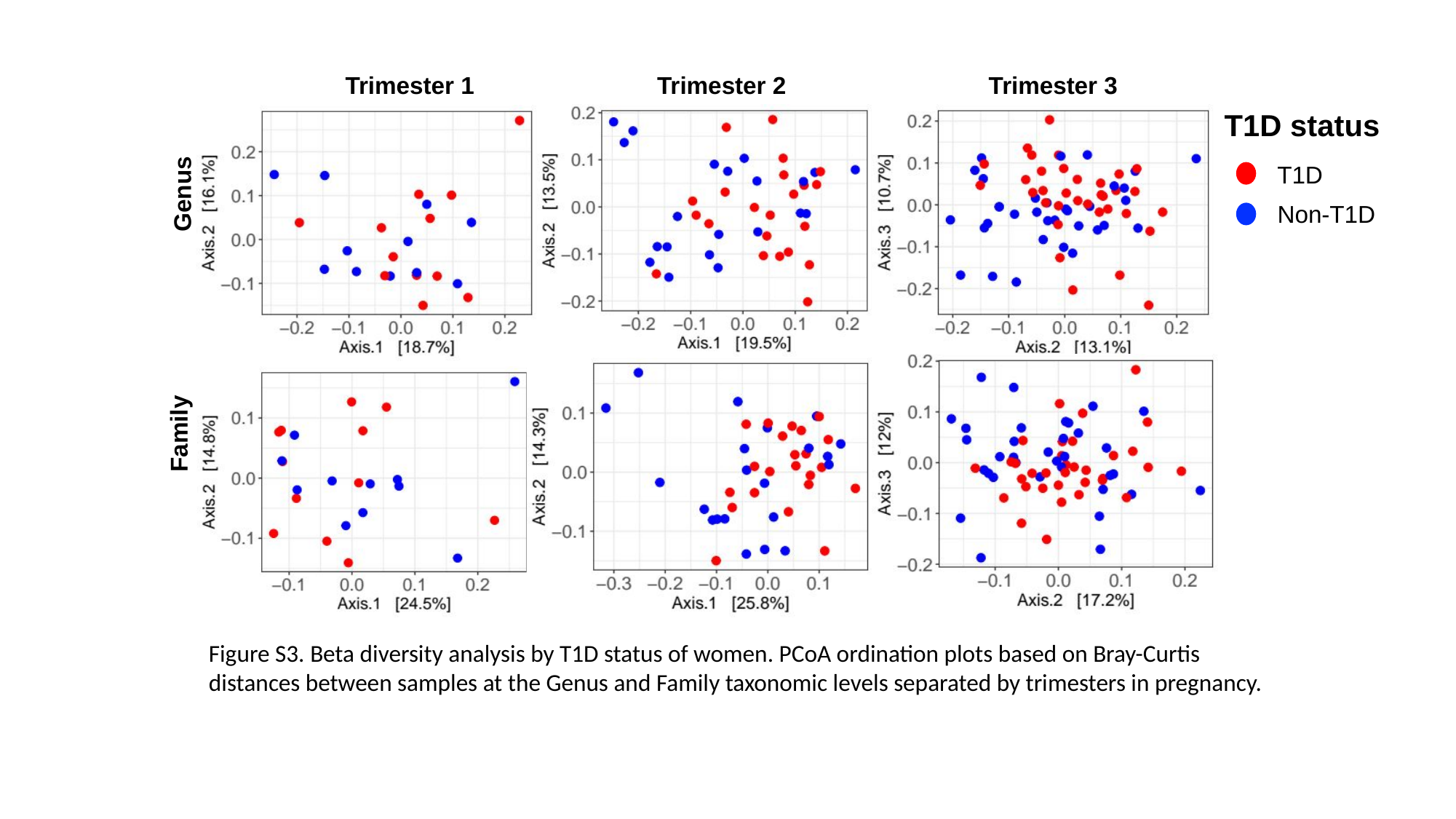

Trimester 1
Trimester 3
Trimester 2
Genus
Family
T1D status
T1D
Non-T1D
Figure S3. Beta diversity analysis by T1D status of women. PCoA ordination plots based on Bray-Curtis distances between samples at the Genus and Family taxonomic levels separated by trimesters in pregnancy.

## Slide 5
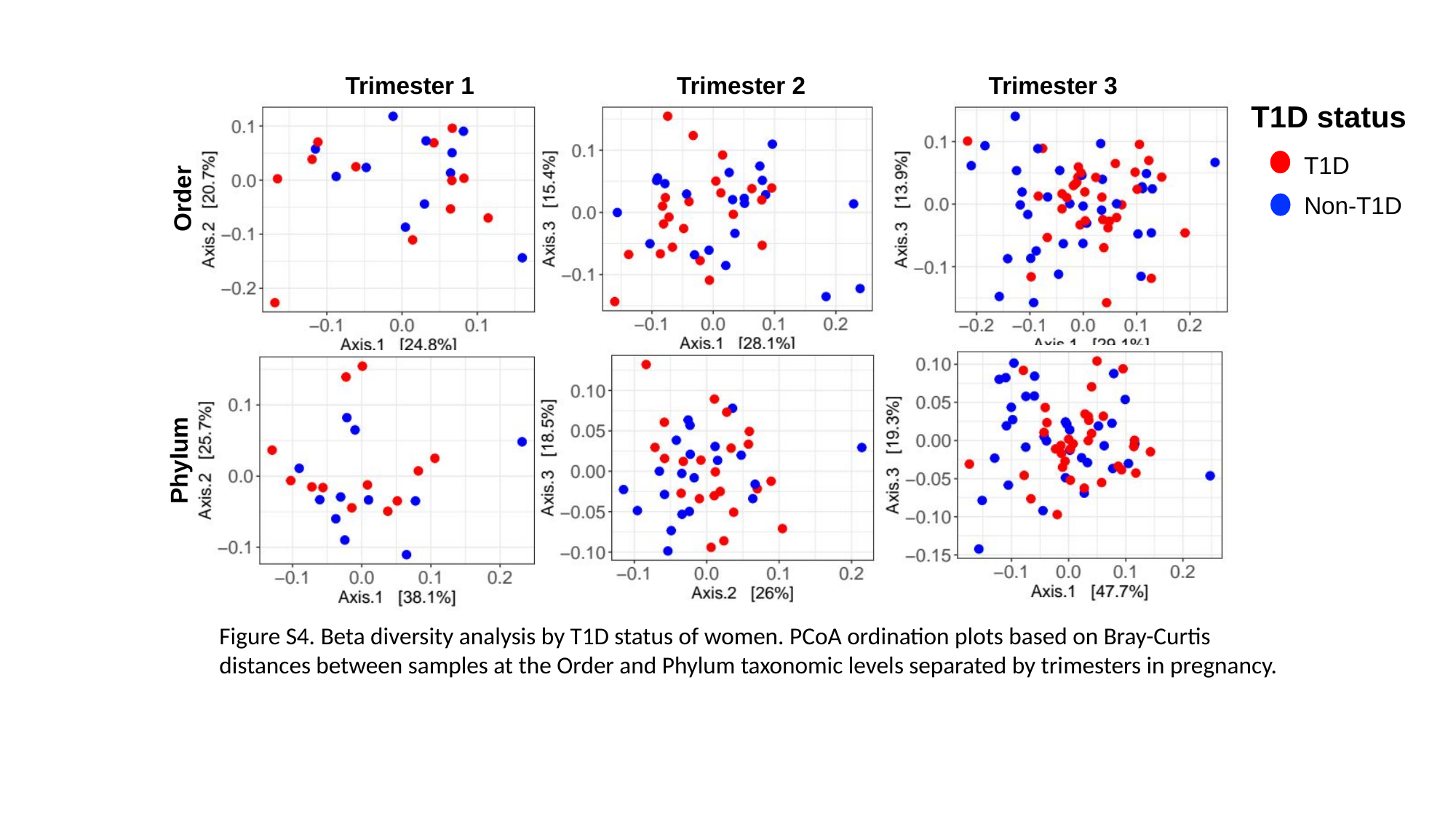

Trimester 1
Trimester 2
Trimester 3
Order
Phylum
T1D status
T1D
Non-T1D
Figure S4. Beta diversity analysis by T1D status of women. PCoA ordination plots based on Bray-Curtis distances between samples at the Order and Phylum taxonomic levels separated by trimesters in pregnancy.

## Slide 6
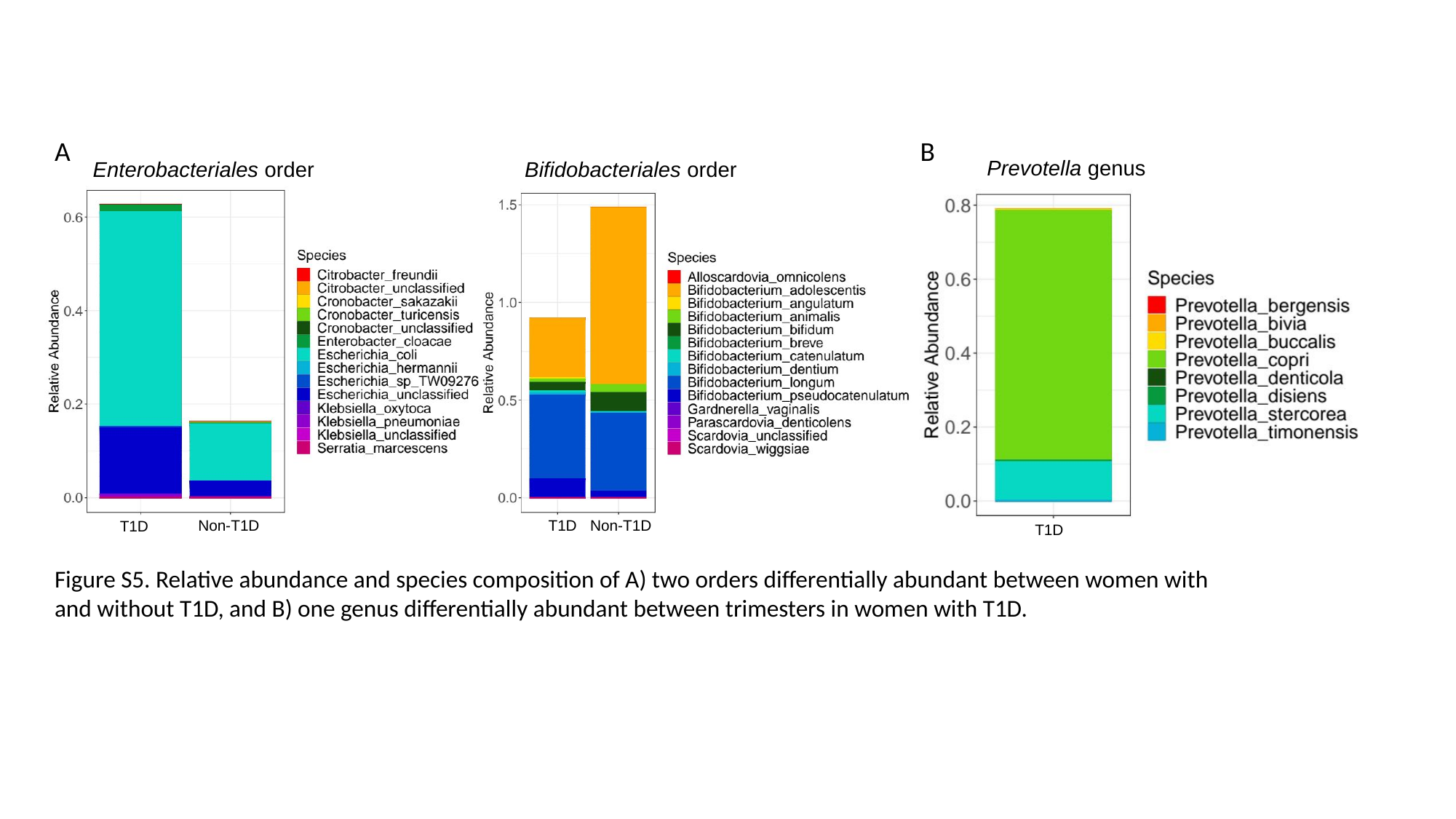

A
B
Prevotella genus
Enterobacteriales order
Non-T1D
T1D
Bifidobacteriales order
Non-T1D
T1D
T1D
Figure S5. Relative abundance and species composition of A) two orders differentially abundant between women with and without T1D, and B) one genus differentially abundant between trimesters in women with T1D.

## Slide 7
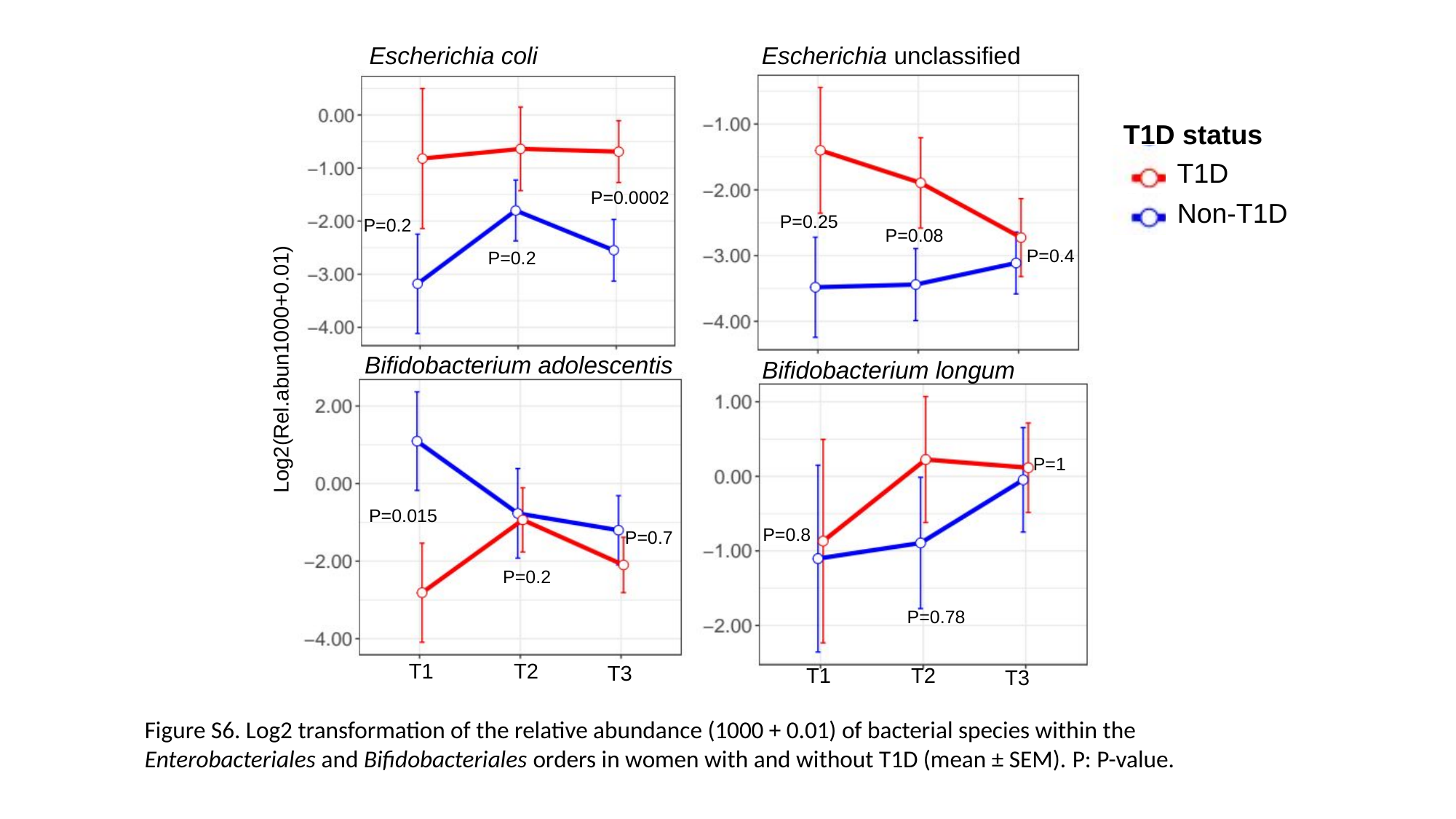

Escherichia coli
Escherichia unclassified
Log2(Rel.abun1000+0.01)
P=0.0002
P=0.25
P=0.2
P=0.08
P=0.4
P=0.2
Bifidobacterium adolescentis
Bifidobacterium longum
P=1
P=0.015
P=0.8
P=0.7
P=0.2
P=0.78
T1
T2
T3
T1
T2
T3
T1D status
T1D
Non-T1D
Figure S6. Log2 transformation of the relative abundance (1000 + 0.01) of bacterial species within the
Enterobacteriales and Bifidobacteriales orders in women with and without T1D (mean ± SEM). P: P-value.

## Slide 8
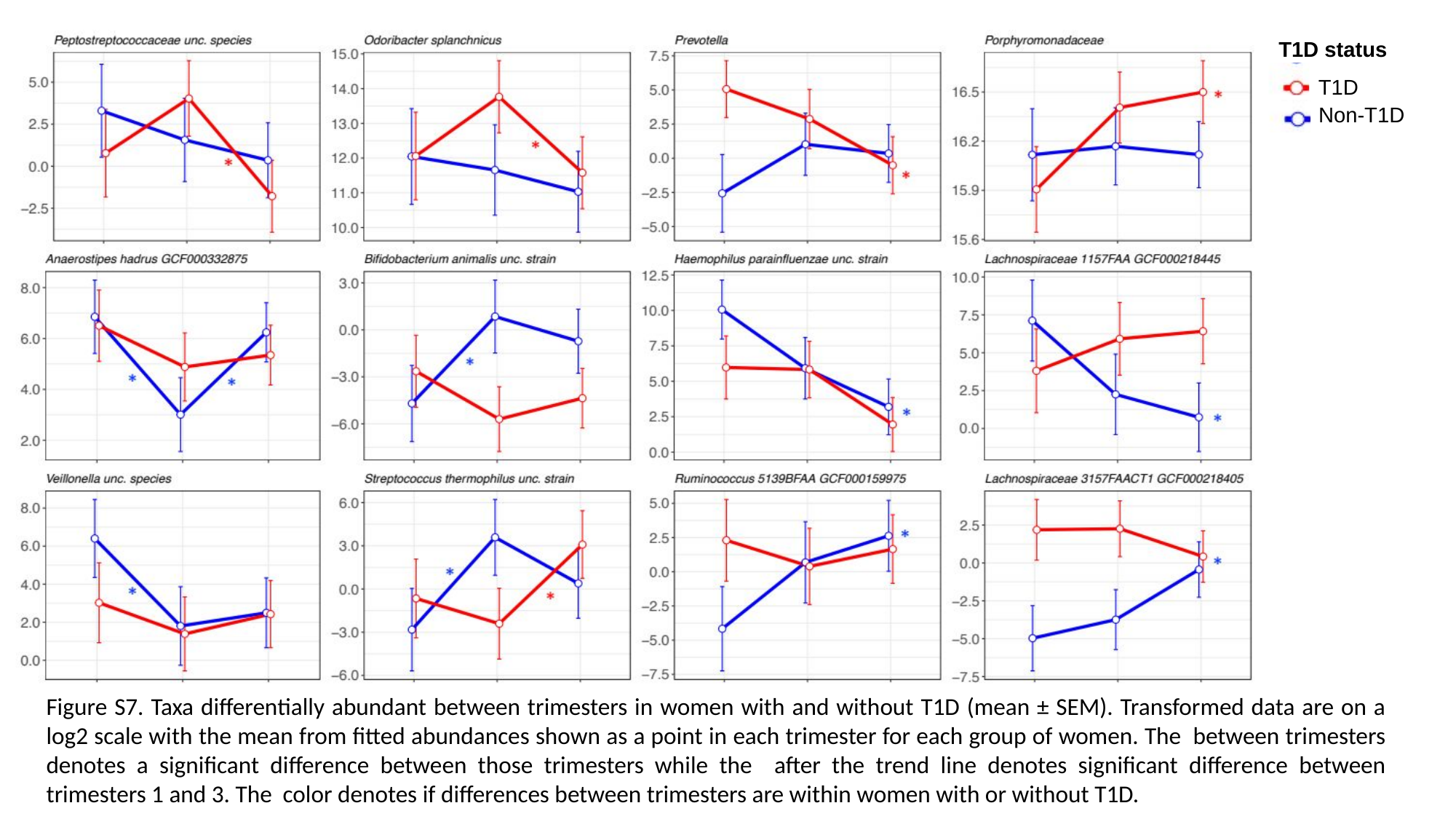

T1D status
T1D
Non-T1D
Figure S7. Taxa differentially abundant between trimesters in women with and without T1D (mean ± SEM). Transformed data are on a log2 scale with the mean from fitted abundances shown as a point in each trimester for each group of women. The between trimesters denotes a significant difference between those trimesters while the after the trend line denotes significant difference between trimesters 1 and 3. The color denotes if differences between trimesters are within women with or without T1D.

## Slide 9
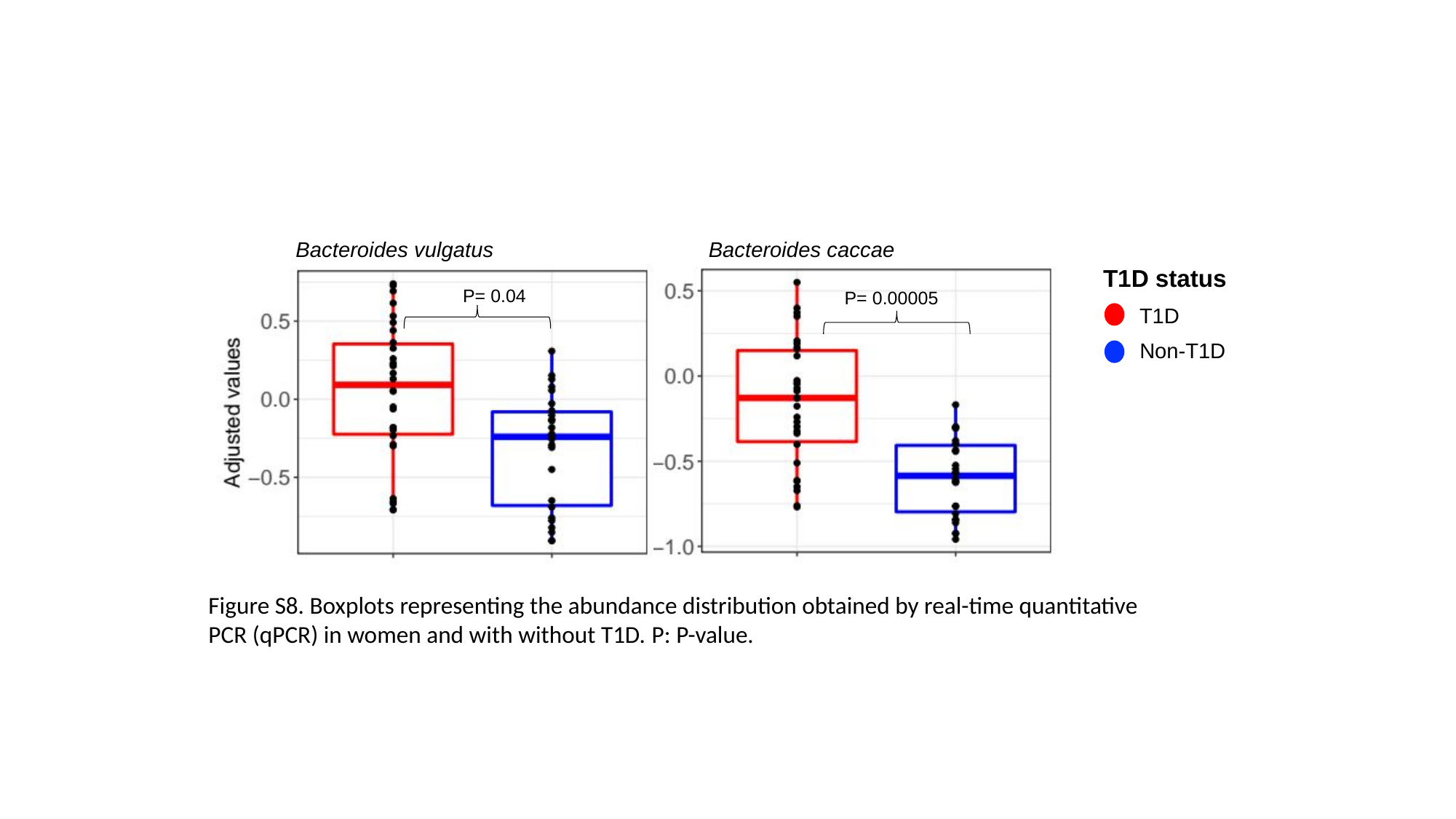

Bacteroides vulgatus
Bacteroides caccae
P= 0.00005
P= 0.04
T1D status
T1D
Non-T1D
Figure S8. Boxplots representing the abundance distribution obtained by real-time quantitative PCR (qPCR) in women and with without T1D. P: P-value.

## Slide 10
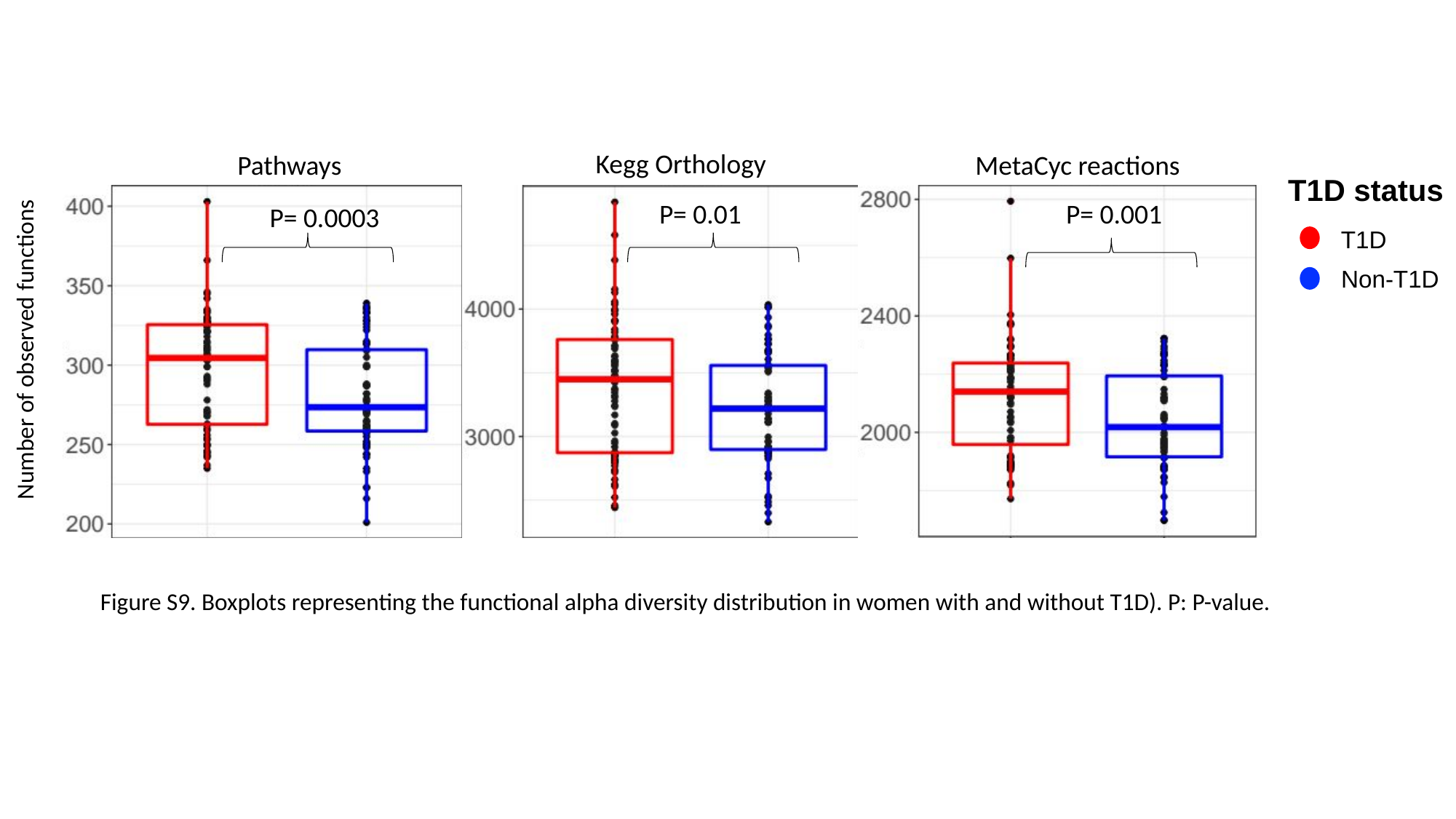

Kegg Orthology
Pathways
MetaCyc reactions
T1D status
T1D
Non-T1D
P= 0.01
P= 0.001
P= 0.0003
Number of observed functions
Figure S9. Boxplots representing the functional alpha diversity distribution in women with and without T1D). P: P-value.

## Slide 11
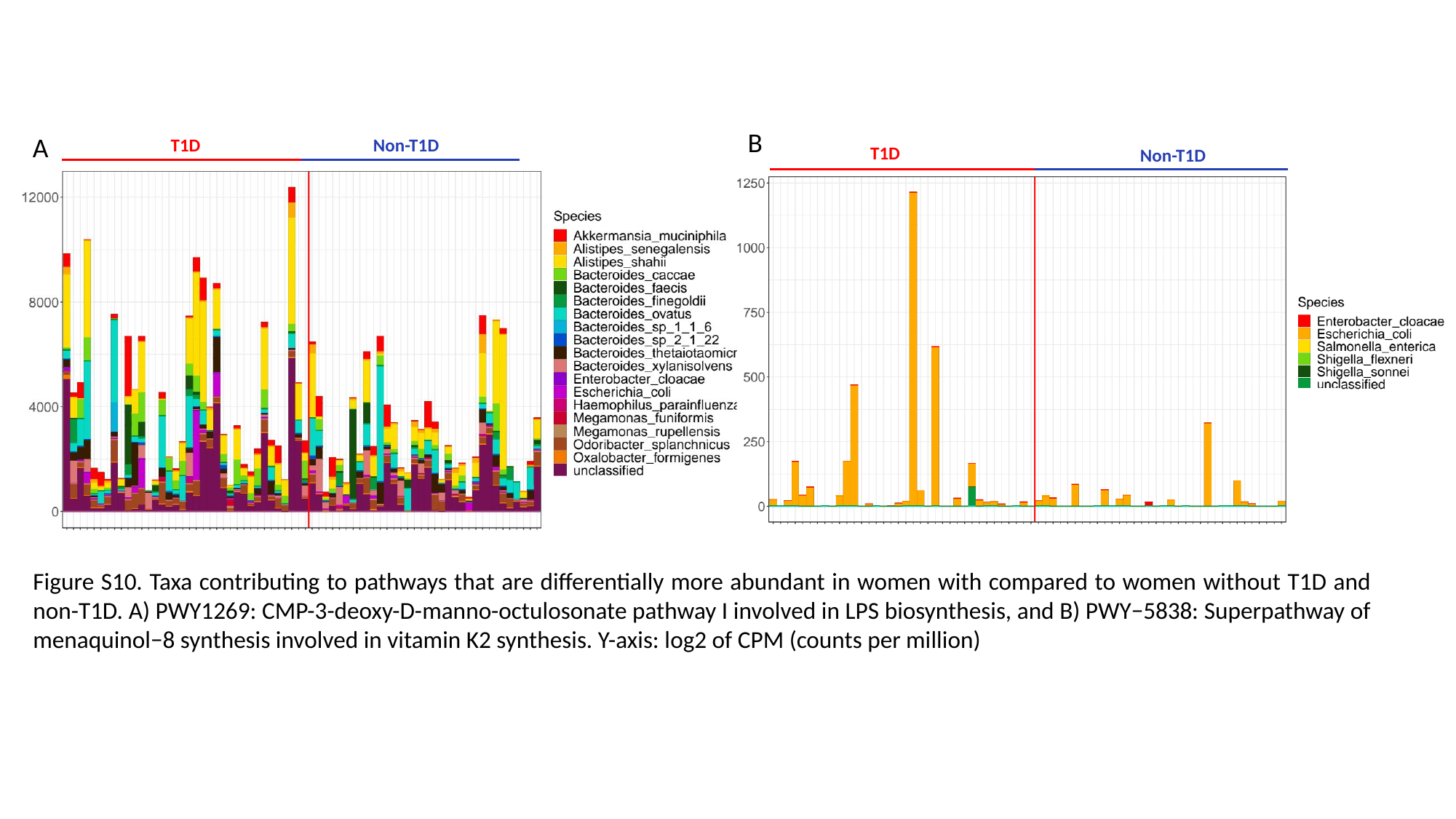

B
T1D
Non-T1D
A
T1D
Non-T1D
Figure S10. Taxa contributing to pathways that are differentially more abundant in women with compared to women without T1D and non-T1D. A) PWY1269: CMP-3-deoxy-D-manno-octulosonate pathway I involved in LPS biosynthesis, and B) PWY−5838: Superpathway of menaquinol−8 synthesis involved in vitamin K2 synthesis. Y-axis: log2 of CPM (counts per million)

## Slide 12
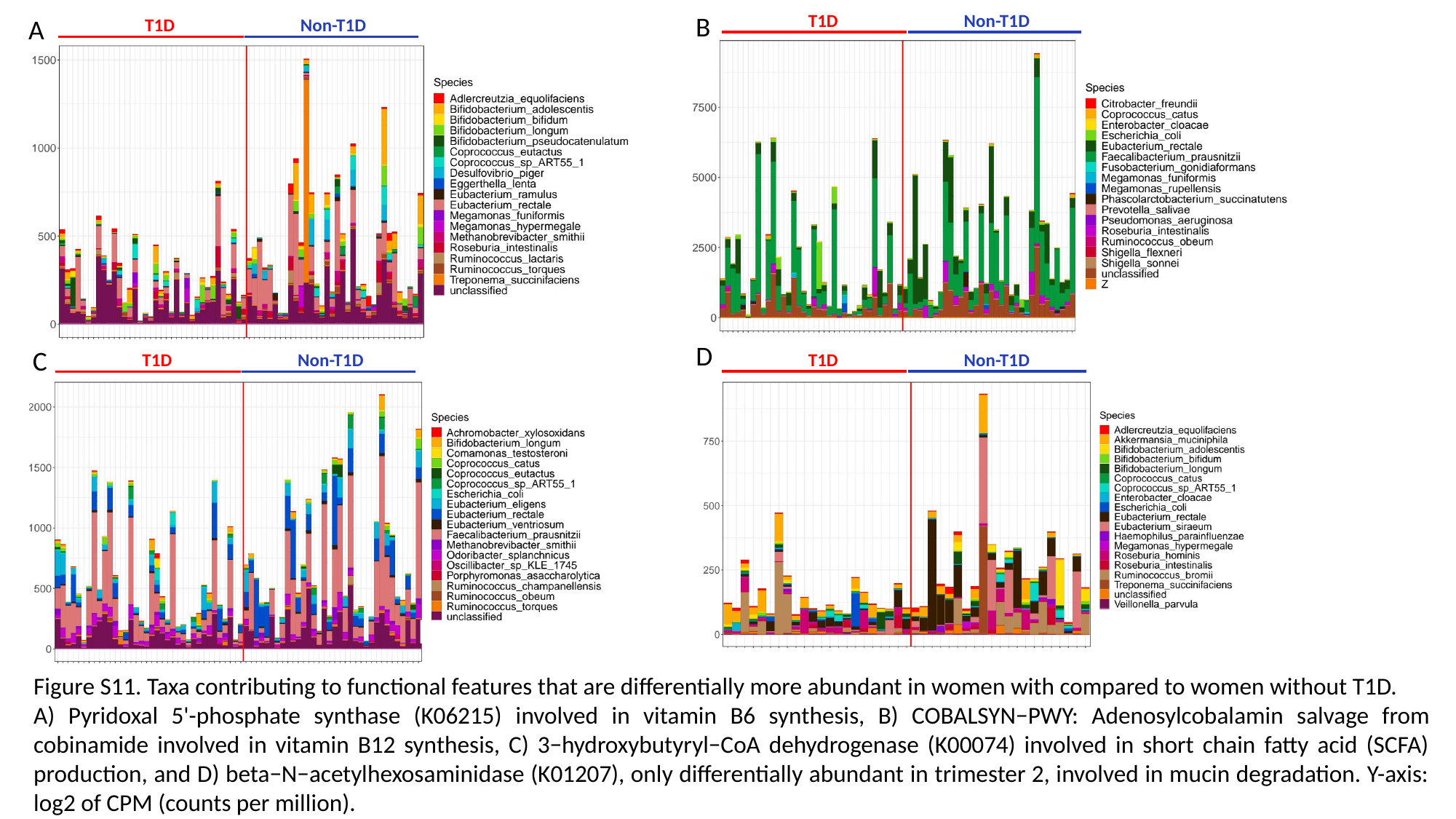

T1D
Non-T1D
B
A
T1D
Non-T1D
D
C
T1D
Non-T1D
T1D
Non-T1D
Figure S11. Taxa contributing to functional features that are differentially more abundant in women with compared to women without T1D.
A) Pyridoxal 5'-phosphate synthase (K06215) involved in vitamin B6 synthesis, B) COBALSYN−PWY: Adenosylcobalamin salvage from cobinamide involved in vitamin B12 synthesis, C) 3−hydroxybutyryl−CoA dehydrogenase (K00074) involved in short chain fatty acid (SCFA) production, and D) beta−N−acetylhexosaminidase (K01207), only differentially abundant in trimester 2, involved in mucin degradation. Y-axis: log2 of CPM (counts per million).

## Slide 13
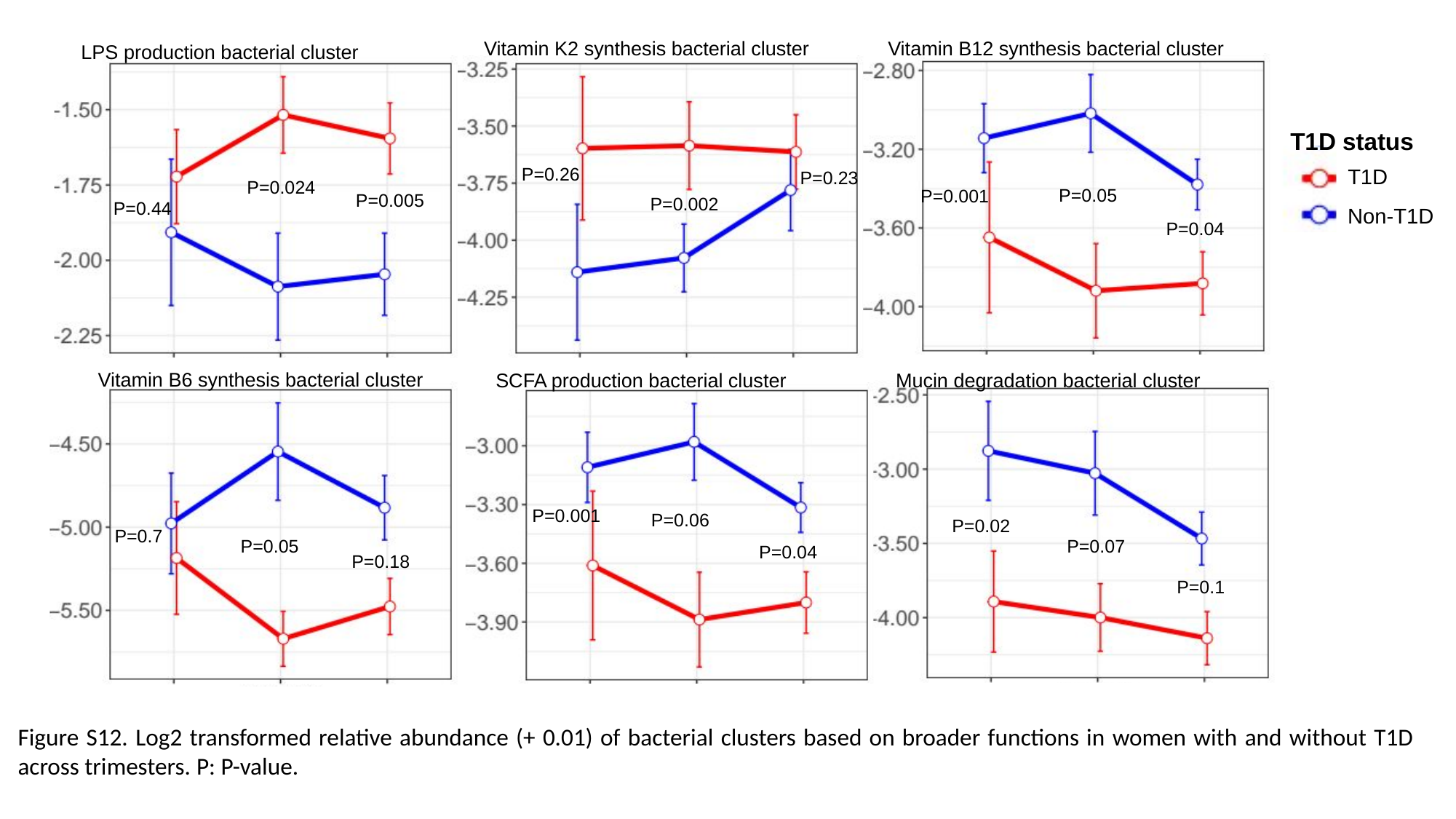

Vitamin K2 synthesis bacterial cluster
P=0.26
P=0.23
P=0.002
Vitamin B12 synthesis bacterial cluster
P=0.05
P=0.001
P=0.04
LPS production bacterial cluster
P=0.024
P=0.005
P=0.44
Vitamin B6 synthesis bacterial cluster
P=0.7
P=0.05
P=0.18
Mucin degradation bacterial cluster
SCFA production bacterial cluster
P=0.001
P=0.06
P=0.04
P=0.02
P=0.07
P=0.1
T1D status
T1D
Non-T1D
Figure S12. Log2 transformed relative abundance (+ 0.01) of bacterial clusters based on broader functions in women with and without T1D across trimesters. P: P-value.

## Slide 14
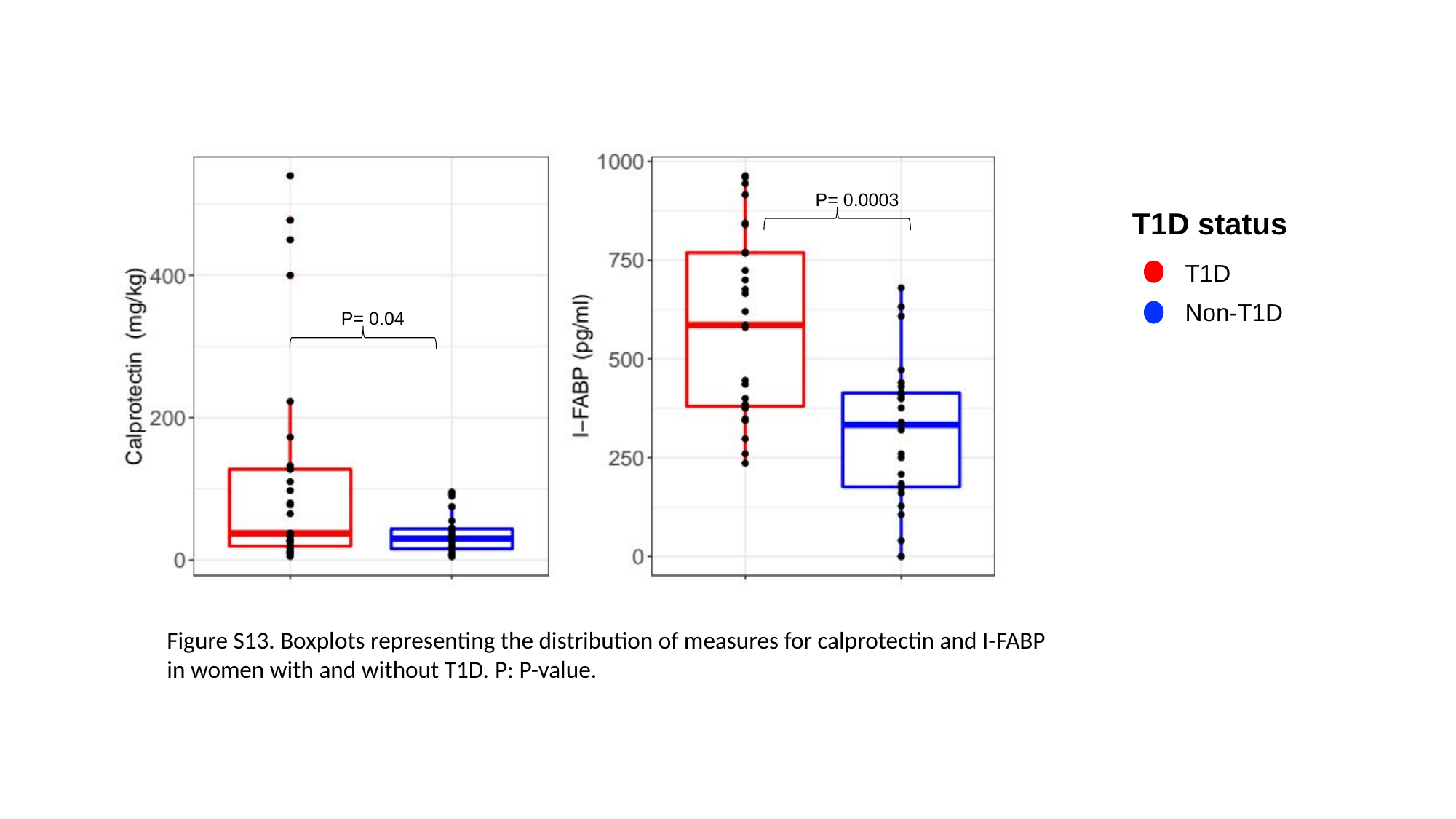

P= 0.0003
T1D status
T1D
Non-T1D
P= 0.04
Figure S13. Boxplots representing the distribution of measures for calprotectin and I-FABP in women with and without T1D. P: P-value.
